# A Floquet engineering approach to optimize Schottky junction-based surface plasmonic waveguides

**DOI:** 10.1038/s41598-023-37801-x

**Published:** 2023-07-02

**Authors:** Kosala Herath, Sarath D. Gunapala, Malin Premaratne

**Affiliations:** 1 Advanced Computing and Simulation Laboratory (AχL), Department of Electrical and Computer Systems Engineering, Monash University, Clayton, VIC 3800 Australia; 2 Jet Propulsion Laboratory, California Institute of Technology, Pasadena, CA 91109 USA

**Keywords:** Electronic devices, Electrical and electronic engineering, Nanophotonics and plasmonics

## Abstract

The ability to finely control the surface plasmon polariton (SPP) modes of plasmonic waveguides unveils many potential applications in nanophotonics. This work presents a comprehensive theoretical framework for predicting the propagation characteristics of SPP modes at a Schottky junction exposed to a dressing electromagnetic field. Applying the general linear response theory towards a periodically driven many-body quantum system, we obtain an explicit expression for the dielectric function of the dressed metal. Our study demonstrates that the dressing field can be used to alter and fine-tune the electron damping factor. By doing so, the SPP propagation length could be controlled and enhanced by appropriately selecting the intensity, frequency and polarization type of the external dressing field. Consequently, the developed theory reveals an unexplored mechanism for enhancing the SPP propagation length without altering other SPP characteristics. The proposed improvements are compatible with existing SPP-based waveguiding technologies and could lead to breakthroughs in the design and fabrication of state-of-the-art nanoscale integrated circuits and devices in the near future.

## Introduction

Meeting the ongoing demand for faster information sharing and data processing through traditional electronic engineering approaches, which are fundamental to all modern electronic devices and components, may be difficult. The thermal and resistor-capacitor time delays pose a challenge in achieving faster and more power-efficient electronic devices^1^. To overcome this bottleneck, optical waves can be used in place of electronic signals, as optical interconnections can enable high-speed and massive data transmissions. However, the conventional photonic engineering approach has poor integration and miniaturization capabilities due to the diffraction limit, which leads to physical size or dimensional inconsistencies between modern electronic and conventional photonic components^2^. To bridge the gap between nanoscale electronic components and microscale photonic components, researchers have turned to plasmonic nanostructures, which have the potential to route and actively manipulate light at the nanoscale^3^. As a result, plasmonics has been investigated in various scientific disciplines, including materials science, chemistry, biology, and communication systems^4,5^.

Surface Plasmon Polaritons (SPPs) are quasiparticles that carry information in plasmonics. They represent a quantum of propagating charge density oscillation at the metal-dielectric interface in the presence of electromagnetic radiation. SPPs are localized at the interface due to their resonant interaction with free electrons in the metal^6^. SPP-based waveguides are used in modern plasmonic devices to focus and deliver light energy to nanoscale regions. The performance of these waveguides depends on the propagation length and the localization of the field in the SPP mode. Achieving high-performance waveguides based on SPP requires considering the propagation losses imposed by the limitations of signal propagation distance. Various modifications to the metal-dielectric interface have been proposed to improve SPP waveguide performance, including arrayed nanoparticles, sharp metal wedges, nanogrooves, gap waveguides, slot waveguides, and cylindrical waveguides^7–12^. Recently, active waveguiding solutions have been proposed to achieve better SPP propagation length in plasmonic waveguides by providing loss-compensating energy to the SPP waveguide^13–15^. One method for enhancing active SPP performance is substituting conventional dielectric material with a doped semiconductor and using electrical pumping to boost performance^16,17^. There is a great need for a clear theoretical understanding of SPP behavior at the interface between a semiconductor and a metal, known as a Schottky junction.

The behavior of SPPs at a Schottky junction is different from conventional SPPs at dielectric-metal interfaces due to the material properties of the semiconductor before and after the Schottky interface formation. The charge carrier density variation in the space charge region formed during the Schottky junction formation impacts the susceptibility and permittivity functions of the interface, offering the ability to manipulate the SPPs propagate along the Schottky junction^17^. This property has attracted researchers’ attention in modern plasmonics^17,18^. However, previous studies^17,19^ on Schottky junction-based SPPs have been restricted to a junction originating with a lossless metal, which is not suitable for real-world plasmonic waveguides with lossy metallic mediums and SPPs with finite propagation lengths. Therefore, it is critical to understand the fundamental mechanism behind Schottky junction-based SPPs that are based on lossy metals to develop next-generation plasmonic waveguides. The purpose of this study is to theoretically analyze the impact of damping effects on Schottky junction-based SPPs and to suggest a novel analytical approach using Floquet engineering to improve the performance of SPPs modes.

Recent advancements in laser technology have made Floquet engineering an intriguing topic, which uses high-intensity periodic driving to obtain new characteristics of matter that are not accessible in equilibrium states^20^. In quantum many-body systems, time-periodic radiation is a powerful tool that can control material properties on an as-needed basis. The Floquet formalism^21^ is the primary analytical tool used in this area of study, and it can describe the effects of an external field even at high intensities, unlike perturbation techniques. The Floquet formalism allows for better control over material properties, making it an essential tool in the study of quantum many-body systems. Instead of treating quantum particles and electromagnetic fields separately, they should be treated as a single composite quantum system in a non-perturbative approach. This system is commonly referred to as a dressed system, with the high-intensity electromagnetic field acting as the dressing field. While Floquet engineering has found applications in various fields of physics, we will specifically examine its application in plasmonic waveguides. The paper by Herath and Premaratne^22^ discusses how to improve the performance of SPPs in metal-dielectric plasmonic waveguides using Floquet engineering. However, there has been no evaluation of how external radiation affects Schottky junction-based plasmon waveguides. This study uses the Floquet formalism to identify new phases of dressed Schottky junctions and manipulate SPP propagation lengths. This allows us to change the propagation length of Schottky junction-based SPP modes while maintaining their other properties. Previous studies have shown that the Schottky junction bias voltage can manipulate the propagation length of SPP, but this will also change the space charge region and alter other properties of the SPP modes^17,23^. The proposed method in this study, using Floquet engineering, can modify the SPP propagation length based on the dressing field’s intensity, frequency, and polarization type. Thus, we believe that Floquet engineering-based method can be applied to nanoplasmonic circuits^24^ in a very promising manner. For this work, it is essential to assume that the system is in a low-temperature stable state, and the electromagnetic field should be solely dressing to prevent any energy exchange with electrons. The electrons in a metallic system can absorb the field through two mechanisms, namely, electron transitions between different energy levels and transitions between various states within the broadened energy level. To avoid these mechanisms, the frequency of the dressing field should be carefully selected to be far from the resonant frequency and larger than the scattering-induced energy band broadening. This allows the avoidance of interband and intraband energy absorptions.

This work examines the optical properties of dressed Schottky junctions and proposes a method to enhance their performance. The analysis considers more generalized conditions than previous studies. The Floquet states in a dressed metallic system are examined, and their optical characteristics are studied using the Floquet formalism and liner-response theory. An analytical expression for the susceptibility function of the dressed metallic system is derived. The external dressing field can manipulate the damping factor for charge carriers in the metal. The wave-function solution of the Floquet state in a quantum Floquet system depends on the radiation strength, which enables the possibility to tailor the charge transport and SPP characteristics under external radiation. The dispersion relations for possible SPP modes in a dressed Schottky junction are derived, and an analytical technique is proposed to reduce electron-impurity scattering in the dressed metal and manipulate the propagation length of SPP modes. A detailed numerical analysis of the Schottky junction-based dressed SPP properties is performed under differing polarization conditions. This research provides a new possibility for controlling the properties of SPP modes within a Schottky junction, which could benefit next-generation nanoplasmonic components and devices.

## Theoretical formulation

The theoretical basis for Schottky junction-based SPPs and their behavior when exposed to a high-intensity dressing field is described in this section. The optical response of a dressed metal is analyzed, and expressions for its susceptibility and dielectric functions are derived. The Floquet–Fermi golden rule is used to examine the damping effects in dressed metal electrons, demonstrating the possibility of manipulating the metal dielectric function. Ultimately, expressions for the dispersion relations of potential SPP modes at a lossy Schottky junction are derived.

### Dielectric function of dressed metals

This part of the analysis presents the derivation of an expression for the conductivity of a dressed metal film. The equilibrium linear response theory is extended to a driven system out of equilibrium, which is probed by a weak external potential. Moreover, an expression for the current response is derived, which depends not only on the frequency of the probe but also on the frequency of the drive. Finally, an analytical expression for the susceptibility and dielectric functions of a dressed metal is presented.

#### Floquet theory

We consider a metal film subjected to a high-intensity periodical dressing field. The wave function of a single electron in the dressed metal can be identified as Floquet states^22,25,26^

1 $$\begin{aligned} {|{\psi _{\alpha }(t)}\rangle } = \exp \left( -i\frac{\epsilon _{\alpha }}{\hbar } t\right) {|{u_{\alpha }(t)}\rangle }. \end{aligned}$$

In this case, $\alpha$ represents a discrete set of quantum numbers, ${|{u_{\alpha }(t)}\rangle }$ are corresponding Floquet modes, and $\epsilon _{\alpha }$ are the corresponding quasienergies. The periodicity of the Floquet modes, allow to rewrite them as a Fourier expansion as follows

2 $$\begin{aligned} {|{u_{\alpha }(t)}\rangle } = \sum _{n} \exp (-in\Omega t) {|{u_{\alpha }^n}\rangle }, \end{aligned}$$

where $\Omega$ is the angular frequency of the dressing field.

#### The Floquet picture of the Kubo formula

In linear response theory, the average current response of an equilibrium system for a probe bias with a vector potential ${\textbf {A}}({\textbf {k}},\omega )$ can be expressed by the general Kubo formula in ${\textbf {k}}$-momentum space^27^

3 $$\begin{aligned} \begin{aligned} {\langle {{\hat{J}}^a({\textbf {k}},\omega )}\rangle }{} & {} = \lim _{\mu \rightarrow 0^+} -\frac{1}{2\pi \hbar V} \sum _{b} \int _{-\infty }^{\infty } \int _{-\infty }^{\infty } \int _{-\infty }^{\infty } \bigg \langle { [{\hat{j}}^a({\textbf {k}},t),{\hat{j}}^b(-{\textbf {k}},t')]\bigg \rangle }_0 A^b({\textbf {k}},t') \exp (i\omega t) \frac{\exp [-i\omega ' (t-t')]}{\omega ' + i\mu } ~ {\textrm{d}}\omega ' {\textrm{d}}t'{\textrm{d}}t \\{} & {} - \frac{e^2n_m}{m} A^a({\textbf {k}},\omega ). \end{aligned} \end{aligned}$$

Here, ${\hat{j}}$ is the single-particle current operator of the system, $\omega$ is the frequency of the response current, *V* is the system volume, $a,b \in \{x,y,z\}$ present the directional components in three-dimensional coordinate space, and ${\langle {\cdot }\rangle }_0$ denotes the statistical thermal average with respect to the system without the probe bias. Due to the presence of high-intensity external dressing in our case, the system will not be in equilibrium. Since the occupation number of Floquet states are independent, we can assume our system to be in a stationary state under the dressing field^28^. From second quantization formalism, we can expand the current operators ${\hat{j}}^{a,b}({\textbf {k}},t)$ using the Floquet states as the basis for our system as follows^29^

4 $$\begin{aligned} {\hat{j}}^a({\textbf {k}},t) = \sum _{\alpha \beta } {\hat{j}}^a_{\alpha \beta }({\textbf {k}},t) {\hat{a}}_{\alpha }^{\dagger }(t_0 = 0){\hat{a}}_{\beta }(t_0 = 0), \end{aligned}$$

where ${\hat{a}}_{\alpha }^{\dagger }(t)$ and ${\hat{a}}_{\alpha }(t)$ are creation and annihilation operators for $\alpha$-th Floquet state. These operators should satisfy the following relationships

5 $$\begin{aligned} {\hat{a}}_{\alpha }^{\dagger } (t){|{0}\rangle } = {|{\psi _{\alpha }(t)}\rangle }, \quad {\hat{a}}_{\alpha }(t){|{0}\rangle } = 0,\quad [{\hat{a}}_{\alpha }(t),{\hat{a}}_{\beta }^{\dagger }(t)]_{\pm } = \delta _{\alpha \beta }, \quad \text {and} \quad [{\hat{a}}_{\alpha }(t),{\hat{a}}_{\beta }(t)]_{\pm } = [{\hat{a}}_{\alpha }^{\dagger }(t),{\hat{a}}_{\beta }^{\dagger }(t)]_{\pm } = 0. \end{aligned}$$

Here, ${|{0}\rangle }$ is the vacuum state containing no particle, and positive (negative) subscripts refer to the fermionic anticommutators (commutators) for the Floquet state particles. Furthermore, ${\hat{j}}^a_{\alpha \beta }({\textbf {k}},t)$ is the single particle matrix element given in Schrödinger’s picture by

6 $$\begin{aligned} {\hat{j}}^a_{\alpha \beta }({\textbf {k}},t) = \langle {\psi _{\alpha }(t)}|{{\hat{j}}^a({\textbf {k}})}|{\psi _{\beta }(t)}\rangle = \langle {u_{\alpha }(t)} |{\exp \left( \frac{i\epsilon _{\alpha }t}{\hbar }\right) {\hat{j}}^a({\textbf {k}}) \exp \left( -\frac{i\epsilon _{\beta }t}{\hbar }\right) }|{u_{\beta }(t)}\rangle , \end{aligned}$$

where ${\hat{j}}^a({\textbf {k}}) = {\hat{j}}^a({\textbf {k}},t=0)$. Considering the Fourier series expand of the time-periodic Floquet modes, we can identify that

7 $$\begin{aligned} {\hat{j}}^a_{\alpha \beta }({\textbf {k}},t) = \sum _{n_1,n_2 = -\infty }^{\infty } \exp \left\{ \frac{i}{\hbar }[(\epsilon _{\alpha } - \epsilon _{\beta }) + (n_1 - n_2)\hbar \Omega ]t\right\} \Big \langle {u_{\alpha }^{n_1}}\Big |{{\hat{j}}^a({\textbf {k}})}\Big |{u_{\beta }^{n_2}}\Big \rangle , \end{aligned}$$

where $n_1,n_2$ are integers. Next, we can evaluate the commutator operator in equation (3) using the above derived second quantization expansion

8 $$\begin{aligned}{}[{\hat{j}}^a({\textbf {k}},t),{\hat{j}}^b(-{\textbf {k}},t')] = \sum _{\alpha \beta } {\hat{j}}^a_{\alpha \beta }({\textbf {k}},t) {\hat{j}}^a_{\beta \alpha }(-{\textbf {k}},t') ({\hat{a}}_{\alpha }^{\dagger }{\hat{a}}_{\alpha } - {\hat{a}}_{\beta }^{\dagger }{\hat{a}}_{\beta }). \end{aligned}$$

Here, ${\hat{a}}_{\alpha }^{\dagger }{\hat{a}}_{\alpha }$ represent the number operator for the $\alpha$-th Floquet state. Thus, we introduce the distribution functions for the Floquet state particles in the system as follows

9 $$\begin{aligned} {\mathscr {F}}_{\alpha } {:}{=}{\langle {{\hat{a}}_{\alpha }^{\dagger }{\hat{a}}_{\alpha }}\rangle }, \quad \text {and} \quad {\mathscr {F}}_{\beta } {:}{=}\big \langle {{\hat{a}}_{\beta }^{\dagger }{\hat{a}}_{\beta }\big \rangle }. \end{aligned}$$

It is important to note that these distribution functions not necessarily to be equilibrium distribution functions, however it is assumed that these are time-independent. Thus, the statistical thermal expectation value of the above commutator becomes

10 $$\begin{aligned} \begin{aligned} \big \langle { [{\hat{j}}^a({\textbf {k}},t),{\hat{j}}^b(-{\textbf {k}},t')]\big \rangle }_0&= \sum _{\alpha \beta } \sum _{n_1, \ldots ,n_4 = -\infty }^{\infty } \exp \left\{ \frac{i}{\hbar }[(\epsilon _{\alpha } - \epsilon _{\beta }) + (n_1 - n_2)\hbar \Omega ]t\right\} \exp \left\{ \frac{i}{\hbar }[(\epsilon _{\beta } - \epsilon _{\alpha }) + (n_3 - n_4)\hbar \Omega ]t'\right\} \\&\quad \times \big \langle {u_{\alpha }^{n_1}}\big |{{\hat{j}}^a({\textbf {k}})}\big |{u_{\beta }^{n_2}}\big \rangle \,\, \big \langle {u_{\beta }^{n_3}}\big |{{\hat{j}}^b(-{\textbf {k}})}\big |{u_{\alpha }^{n_4}}\big \rangle ({\mathscr {F}}_{\alpha } - {\mathscr {F}}_{\beta }). \end{aligned} \end{aligned}$$

Then, we can represent the vector potential corresponding to the probe bias in the frequency domain as follows

11 $$\begin{aligned} A^b({\textbf {k}},t') = \frac{1}{2\pi } \int _{-\infty }^{\infty } A^b({\textbf {k}},\omega '')\exp (-i\omega ''t') ~{\textrm{d}}\omega '', \end{aligned}$$

and submitting these back into the Kubo formula and evaluating the time integrals and $\omega '$ integral, we can obtain

12 $$\begin{aligned} \begin{aligned} \langle {{\hat{J}}^a({\textbf {k}},\omega )}\rangle&= \lim _{\mu \rightarrow 0^+} -\frac{1}{\hbar V} \sum _{b} \int _{-\infty }^{\infty } \sum _{\alpha \beta } \sum _{n_1, \ldots ,n_4 = -\infty }^{\infty } \frac{\delta \left( \omega - \omega '' + (n_1 - n_2 + n_3 -n_4)\Omega \right) }{\left[ \omega + \frac{1}{\hbar }(\epsilon _{\alpha } - \epsilon _{\beta }) + (n_1 - n_2)\Omega + i\mu \right] }\\&\quad \times \big \langle {u_{\alpha }^{n_1}}\big |{{\hat{j}}^a({\textbf {k}})}\big |{u_{\beta }^{n_2}}\big \rangle \,\,\big \langle {u_{\beta }^{n_3}}\big |{{\hat{j}}^b(-{\textbf {k}})}\big |{u_{\alpha }^{n_4}}\big \rangle A^b({\textbf {k}},\omega '') \left( {\mathscr {F}}_{\alpha } - {\mathscr {F}}_{\beta }\right) ~{\textrm{d}}\omega '' - \frac{e^2n_m}{m} A^a({\textbf {k}},\omega ). \end{aligned} \end{aligned}$$

We can specify the probe electric field relation with vector potential using the electromagnetic theory

13 $$\begin{aligned} A^b({\textbf {k}},\omega ) = -\frac{i}{(\omega + i\gamma )} E^b({\textbf {k}},\omega ). \end{aligned}$$

Here, the factor $\gamma$ is a phenomenological way to include electron scattering-caused damping effects in the quantum calculations. Then, we can rewrite the Kubo formula in a compact form

14 $$\begin{aligned} \langle {{\hat{J}}^a({\textbf {k}},\omega )}\rangle = \sum _{b} \int _{-\infty }^{\infty } \sigma ^{ab}({\textbf {k}},\omega ,\omega '') E^b({\textbf {k}},\omega '') ~{\textrm{d}}\omega '', \end{aligned}$$

by initiating the conductivity tensor for the dressed metallic system

15 $$\begin{aligned} \begin{aligned} \sigma ^{ab}({\textbf {k}},\omega ,\omega '')&= \lim _{\mu \rightarrow 0^+} \frac{i}{\hbar V} \sum _{\alpha \beta } \sum _{n_1, \ldots ,n_4 = -\infty }^{\infty } \frac{\delta \left( \omega - \omega '' + (n_1 - n_2 + n_3 -n_4)\Omega \right) }{\left[ \omega + \frac{1}{\hbar }(\epsilon _{\alpha } - \epsilon _{\beta }) + (n_1 - n_2)\Omega + i\mu \right] }\\&\quad \times \big \langle {u_{\alpha }^{n_1}}\big |{{\hat{j}}^a({\textbf {k}})}\big |{u_{\beta }^{n_2}}\big \rangle \,\, \big \langle {u_{\beta }^{n_3}}\big |{{\hat{j}}^b(-{\textbf {k}})}\big |{u_{\alpha }^{n_4}}\big \rangle \frac{\left( {\mathscr {F}}_{\alpha } - {\mathscr {F}}_{\beta }\right) }{\omega + i\gamma }+ \frac{ie^2n_m}{m(\omega + i\gamma )} \delta (\omega - \omega '')\delta _{ab}, \end{aligned} \end{aligned}$$

Based on this expression, the current is no longer a simple product of conductivity and perturbation electric field as it is convoluted over the bias frequency $\omega ''$, as opposed to the un-driven case. There is significance in the fact that the conductivity tensor depends on both the response frequency $\omega$ and the bias frequency $\omega ''$.

With the above-derived general expression for conductivity, we can determine a new expression for our Floquet system by specifying the conditions which affect the parameters. First, we assume that the response and bias frequency $\omega$ and $\omega ''$ are in the central Floquet zone

16 $$\begin{aligned} |\omega |,|\omega ''|< \left| {\Omega }/{2}\right| \quad \Rightarrow \quad |\omega -\omega ''| < \Omega . \end{aligned}$$

Under this condition, we can identify that the delta distribution in Eq. (15) only can be non-zero with the condition

17 $$\begin{aligned} n_1 - n_2 + n_3 - n_4 = 0. \end{aligned}$$

Focusing on more special case of conductivity by analyzing only the longitudinal conductivity where $a = b = x$, and assuming that the current response is spatially homogeneous (${\textbf {k}} \rightarrow 0$), we can obtain

18 $$\begin{aligned} \begin{aligned} \sigma ^{xx}(\omega )&= \lim _{\mu \rightarrow 0^+} \frac{i}{\hbar V(\omega + i\gamma )} \frac{e^2}{m^2} \sum _{\alpha \beta } \sum _{n_1, \ldots ,n_4 = -\infty }^{\infty } \frac{\left( {\mathscr {F}}_{\alpha } - {\mathscr {F}}_{\beta }\right) }{\left[ \omega + \frac{1}{\hbar }(\epsilon _{\alpha } - \epsilon _{\beta }) + (n_1 - n_2)\Omega + i0^+\right] } \big \langle {u_{\alpha }^{n_1}}\big |{{\hat{p}}^x}\big |{u_{\beta }^{n_2}}\big \rangle \,\, \big \langle {u_{\beta }^{n_3}}\big |{{\hat{p}}^x}\big |{u_{\alpha }^{n_4}}\big \rangle \\&\quad + \frac{ie^2n_m}{m(\omega + i\gamma )}, \end{aligned} \end{aligned}$$

where ${\hat{p}}^x$ is the *x*-directional momentum operator. Polarization and current responses are physically indistinguishable effects of the conductivity tensor. Accordingly, susceptibility function of the system can be expressed in general as a function of linear conductivity^30^

19 $$\begin{aligned} \chi ^{xx} (\omega ) = \frac{\sigma ^{xx}(\omega )}{-i\omega }. \end{aligned}$$

Subsequently, we can identify the susceptibility function of the dressed quantum system as

20 $$\begin{aligned} \begin{aligned} \chi ^{xx} (\omega )&= \lim _{\mu \rightarrow 0^+} \frac{1}{\hbar \omega (\omega + i\gamma )} \frac{e^2}{m^2V} \sum _{\alpha \beta } \sum _{n_1, \ldots ,n_4 = -\infty }^{\infty } \frac{\left( {\mathscr {F}}_{\alpha } - {\mathscr {F}}_{\beta }\right) }{\left[ \omega + \frac{1}{\hbar }(\epsilon _{\alpha } - \epsilon _{\beta }) + (n_1 - n_2)\Omega + i\mu \right] } \big \langle {u_{\alpha }^{n_1}}\big |{{\hat{p}}^x}\big |{u_{\beta }^{n_2}}\big \rangle \,\, \big \langle {u_{\beta }^{n_3}}\big |{{\hat{p}}^x}\big |{u_{\alpha }^{n_4}}\big \rangle \\&\quad + \frac{e^2n_m}{m\omega (\omega + i\gamma )}. \end{aligned} \end{aligned}$$

The study involves a dressed metallic system that utilizes the free electron model to explain its transport properties. The particle distribution functions can be described using the Fermi–Dirac distribution. Additionally, very low-temperature conditions ($T \rightarrow 0$) are considered, leading to a re-writing of the Fermi–Dirac distribution

21 $$\begin{aligned} {\mathscr {F}}(\epsilon ) = \lim _{T \rightarrow 0} \frac{1}{\exp (\frac{\epsilon - \epsilon _F}{k_B T})+1} \approx \Theta (\epsilon _F - \epsilon ), \end{aligned}$$

where, $\epsilon _F$ is the Fermi energy, $k_B$ is the Boltzmann constant, *T* is the absolute temperature, and ${\Theta}(\cdot )$ is the Heaviside function. Now, we can restructure the general susceptibility function according to the dressed metallic system

22 $$\begin{aligned} \begin{aligned} \chi ^{xx} (\omega )&= \lim _{\mu \rightarrow 0^+} \frac{1}{\hbar \omega (\omega + i\gamma )} \frac{e^2}{m^2V} \sum _{\alpha \beta } \sum _{n_1, \ldots ,n_4 = -\infty }^{\infty } \frac{\left( \Theta (\epsilon _F - \epsilon _{\alpha })- \Theta (\epsilon _F - \epsilon _{\beta })\right) }{\left[ \omega + \frac{1}{\hbar }(\epsilon _{\alpha } - \epsilon _{\beta }) + (n_1 - n_2)\Omega + i\mu \right] } \big \langle {u_{\alpha }^{n_1}}\big |{{\hat{p}}^x}\big |{u_{\beta }^{n_2}}\big \rangle \,\, \big \langle {u_{\beta }^{n_3}}\big |{{\hat{p}}^x}\big |{u_{\alpha }^{n_4}}\big \rangle \\&\quad + \frac{e^2n_m}{m\omega (\omega + i\gamma )}. \end{aligned} \end{aligned}$$

Considering the transport properties of metallic electrons, it’s only necessary to analyze the behavior of the conduction electrons. This limits the analysis to electrons with energy similar to the Fermi energy. This leads to assume that $\epsilon _{\alpha } = \epsilon '$, and $\epsilon _{\beta } = \epsilon ''$, where $\epsilon ',\epsilon '' \rightarrow \epsilon _F$. Next, we can apply these changes back into the susceptibility function and obtain

23 $$\begin{aligned} \begin{aligned} \chi ^{xx} (\omega )&= \lim _{\mu \rightarrow 0^+} \lim _{\epsilon ',\epsilon '' \rightarrow \epsilon _F} \frac{1}{\hbar \omega (\omega + i\gamma )} \frac{e^2}{m^2V} \sum _{\alpha \beta } \sum _{n_1, \ldots ,n_4 = -\infty }^{\infty } \frac{\left( \Theta (\epsilon _F - \epsilon ')- \Theta (\epsilon _F - \epsilon '')\right) }{\left[ \omega + \frac{1}{\hbar }(\epsilon ' - \epsilon '') + (n_1 - n_2)\Omega + i\mu \right] } \big \langle {u_{\alpha }^{n_1}}\big |{{\hat{p}}^x}\big |{u_{\beta }^{n_2}}\big \rangle \,\, \big \langle {u_{\beta }^{n_3}}\big |{{\hat{p}}^x}\big |{u_{\alpha }^{n_4}}\big \rangle \\&\quad + \frac{e^2n_m}{m\omega (\omega + i\gamma )}. \end{aligned} \end{aligned}$$

In our study, we assume that $\omega \ne 0$ and $\omega < |{\Omega }/{2}|$. It follows that the denominator of the above expression cannot be very small. Therefore, we can see that the first term of the above expression goes to zero. Finally, now we can assume that there is no contribution from the first term in the susceptibility function. Moreover, we can derive final expression for the susceptibility function for a dressed metallic quantum system as

24 $$\begin{aligned} { \chi ^{xx} (\omega ) = \frac{\epsilon _0\omega _{pm}^2}{\omega (\omega + i\gamma )}, \quad \text {where} \quad \omega _{pm} = \sqrt{\frac{e^2n_m}{\epsilon _0 m}}. } \end{aligned}$$

Here, $\gamma _0$ is the un-driven damping factor. Finally, we can identify the dressed metal dielectric function

25 $$\begin{aligned} { \varepsilon _m (\omega ) = \varepsilon _{hm} - \frac{\chi ^{xx} (\omega )}{\epsilon _0} = \varepsilon _{hm} - \frac{\omega _{pm}^2}{\omega (\omega + i\gamma )}, } \end{aligned}$$

where $\varepsilon _{hm}$ is the high-frequency permittivity of the metal. Although the derived expression is the same as the general Drude-Sommerfeld model description, we need to consider the effects on the damping factor $\gamma$ induced by the dressing field. As the previous literature^22,31^ describes, the dressing field modifies the electron wave functions. As long as the electron scattering rate and the electron transport damping factor depend on the wave function, we can control the damping factor as well as the optical properties of the system by applying an external dressing field. These effects can be analytically evaluated by applying the Floquet–Fermi golden rule for a dressed metallic system^22^. This enables us to achieve high-performance SPP modes in Schottky junction-based waveguides. Next, we can rewrite the *x*-directional metal dielectric function by introducing the normalized damping factor ${\tilde{\gamma }}$ as follows

26 $$\begin{aligned} \varepsilon _m (\omega ) = \varepsilon _{hm} - \frac{\omega _{pm}^2}{\omega (\omega + i{\tilde{\gamma }}\gamma _0)}, \quad \text {where} \quad {\tilde{\gamma }} = \frac{\gamma }{\gamma _0}. \end{aligned}$$

Here, $\gamma _0$ is the un-driven damping factor. Moreover, ${\tilde{\gamma }}$ depends on the intensity and the frequency of the applied dressing field^22^. For details on the derivation of these results, see Section 1 and 2 of the Supplementary Information. Under the results section, we investigate numerically how metal dielectric function can be manipulated under various driving fields.

### SPP modes at the Schottky junction

**Figure 1:**
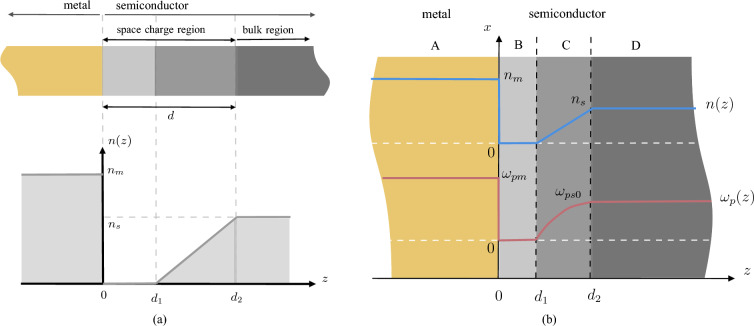
(**a**) Formation of a Schottky junction (top) and piecewise-linear variation of charge density in the Schottky junction (bottom) under the full depletion approximation. Here, $n_s$ is the bulk charge density in the semiconductor, $n_m$ is the charge density in the metal, and *d* represents the width of the space charge region. (**b**) A schematic illustration of the Schottky junction, including divided of the junction regions A, B, C and D are based on the carrier density distribution. Furthermore, carrier density *n*(*z*) and plasma frequency $\omega _P(z)$ for each region are depicted. The plots presented in this figure may not adhere to a linear scale on the vertical axis.

Our primary interest is to analyze the optical properties of Schottky junctions and achieve improved SPP modes at the interface. The interface between a semi-infinite metal and a semi-infinite n-type semiconductor film in the *xy* plane of the Cartesian coordinate space is considered as shown in Fig. 1a top section. The contact between metals and semiconductors leads to the flow of free charge carriers until their Fermi levels align according to thermodynamic principles, causing changes in charge density and creating a space charge region near the interface of the two materials. Notably, the width of the space charge region *d* can be manipulated by applying an external voltage across the interface. Due to this, the Schottky junction plays a crucial role in modern electronics.

In general, a complex charge density profile can be approximated by a piecewise-linear fluctuation. Therefore, to model the charge profile of the semiconductor region, a piecewise-linear variation is used as shown in Fig. 1a bottom section. This model has been used in earlier literature^17,19^, and is also employed in this study for comparison purposes. Additionally, the frequencies of the electromagnetic fields are carefully selected to be in an off-resonant regime to minimize photon absorption by the system.

The complex dielectric functions for n-type semiconductor $\varepsilon _s$ can be described using the following form^32^:

27 $$\begin{aligned} \varepsilon _{s} (\omega ) = \varepsilon _{\text {intra}} (\omega ) + \varepsilon _{\text {inter}} (\omega ) \end{aligned}$$

The proposed approach explicitly distinguishes between the intraband effects $\varepsilon _{\text {intra}}$ and the interband effects $\varepsilon _{\text {inter}}$. In this context, the intraband effects are generated by the free electrons, whereas the interband effects are generated by the bound electrons. The intraband component of the dielectric function is can be characterized by the Drude model^33^

28 $$\begin{aligned} \varepsilon _{\text {intra}} (\omega ) = \varepsilon _{\infty } - \frac{\omega _{p}^2}{\omega ^2+i\omega \varsigma _s}. \end{aligned}$$

Here, $\varepsilon _{\infty }$ is the high-frequency permittivity of the semiconductor, $\omega _{p}$ is the plasma frequency of the semiconductor, and $\varsigma _{s}$ is the damping factor of the semiconductor induced by the electron scattering in the material. The interband component of the dielectric function can be represented by both a real part and an imaginary part:$\varepsilon _{\text {inter}} (\omega ) = \varepsilon '_{\text {inter}} (\omega ) + i\varepsilon ''_{\text {inter}} (\omega )$. Under the absence of interband absorptions we can neglect the imaginary part of the interband component. This leads to

29 $$\begin{aligned} \varepsilon _{s} (\omega ) = \varepsilon _{hs} - \frac{\omega _{p}^2}{\omega ^2+i\omega \varsigma _s}, \end{aligned}$$

where $\varepsilon _{hs} = \varepsilon _{\infty } + \varepsilon '_{\text {inter}} (\omega )$. Generally, $\varepsilon _{hs}$ exhibits dependence on the angular frequency. However, for certain spectral ranges, it can be approximately treated as constant^34^. Since the semiconductor plasma frequency is contingent upon the electron density, the dielectric function is also influenced by variations in electron density. In our system, the electron density of the semiconductor varies with the coordinates along the *z*-direction. Hence, the dielectric function of the semiconductor relies on both the angular frequency of the SPP excitation radiation and the spatial coordinate in the *z*-direction. Therefore, we can determine the dielectric function of the semiconductor in our Schottky junction as follows^35^

30 $$\begin{aligned} \varepsilon _{s} (z,\omega ) = \varepsilon _{hs} \left[ 1 - \frac{\omega _{ps}^2(z)}{\omega ^2+i\omega \varsigma _s} \right] , \quad \text {where} \quad \omega _{ps}(z) = \sqrt{\frac{n(z)e^2}{\varepsilon _{hs} \varepsilon _{0} m}}. \end{aligned}$$

Here, *n*(*z*) is position-dependent free charge carrier density, $\varepsilon _0$ is the vacuum permittivity, and *m* is the effective mass of the free charge carriers. In addition, we assume that the damping factor is not dependent on the free charge carrier density. Moreover, we define the plasma frequency of the bulk semiconductor region as

31 $$\begin{aligned} \omega _{ps0} = \sqrt{ \frac{n_se^2}{\varepsilon _{hs}\varepsilon _0 m}}, \end{aligned}$$

and it is independent of the position. Since the charge density inside the semiconductor of the Schottky junction varies, we can identify four main regions inside a Schottky junction, as depicted in Fig. 1b. For the metal region, we use the general dielectric function expression derived in Eq. (26). With the dressing field removed, one is left with the well-known Drude model-based metal dielectric function expression.Then, we summarize these characteristics of each region under the Table 1.

**Table 1:** Characteristics of each region in the Schottky junction. Here, $r = {1}/{(d_2 -d_1)}$, and $h = -rd_1$.

Region	Range	Charge carrier density *n*(*z*)	Plasma frequency $\omega _p$	Dielectric function $\varepsilon (z,\omega )$
A	$z \le 0$	$n_m$	$\omega _{pm}$	$1 - \frac{\omega _{pm}^2}{\omega ^2 + i \omega {\tilde{\gamma }} \gamma _0}$
B	$0 < z \le d_1$	0	0	$\varepsilon _{hs}$
C	$d_1 < z \le d_2$	$n_s(rz + h)$	$\omega _{ps0} \sqrt{rz + h}$	$\varepsilon _{hs} \left[ 1 - \frac{r\omega _{ps0}^2}{\omega ^2 + i \omega \varsigma _{s}} z + \frac{h\omega _{ps0}^2}{\omega ^2 + i \omega \varsigma _{s}} \right]$
D	$d_2 < z$	$n_s$	$\omega _{ps0}$	${ \varepsilon _{hs} \left[ 1 - \frac{\omega _{ps0}^2}{\omega ^2 + i \omega \varsigma _{s}}\right] }$

Next, we try to identify electromagnetic wave modes ${\textbf {E}}(x,y,z,t)$ and ${\textbf {H}}(x,y,z,t)$ that can exist on the Schottky junction. Here, we represent the electric field of the SPP mode with ${\textbf {E}}$ and the magnetic field with ${\textbf {H}}$. Then, we hope to find solutions with two properties: (i) wave modes propagate through the surface towards the *x*-direction, and (ii) wave modes decay through both mediums in a perpendicular direction to the surface. Without loss of generality, we can assume that $|{\textbf {E}}|$ and $|{\textbf {H}}|$ are independent of *y*-coordinates. Therefore, we can find the solutions for ${\textbf {E}}$ and ${\textbf {H}}$ by defining them as vector components of the Cartesian coordinate system in the following form

32 $$\begin{aligned} {\textbf {E}} (x,y,z,t) = \left( {\mathscr {E}}_x(z)e^{ik_x x - i\omega t },0,{\mathscr {E}}_z(z)e^{ik_x x - i\omega t }\right) ^{\textsf{T}}, \quad \text {and} \quad {\textbf {H}} (x,y,z,t) = \left( 0,{\mathscr {H}}_y(z)e^{ik_x x - i\omega t },0\right) ^{\textsf{T}}. \end{aligned}$$

Here, we restricted our study to the TM-polarization mode. Furthermore, $\omega$ is the frequency of the SPP mode, and $k_x$ represents the *x*-directional wavenumber. Using the well-known Maxwell’s equations, we can derive two differential equations for the possible electromagnetic modes in a Schottky junction

33 $$\begin{aligned} {\mathscr {E}}_x(z) = \frac{i}{k_x} \left[ \frac{\partial {\mathscr {E}}_z(z)}{\partial z} + {\mathscr {E}}_z(z) \frac{\partial \ln {\varepsilon (z,\omega )}}{\partial z} \right] , \end{aligned}$$

and

34 $$\begin{aligned} \begin{aligned} \frac{\partial ^2{\mathscr {E}}_z(z)}{{\partial z}^2} + \frac{\partial \ln {\varepsilon (z,\omega )}}{\partial z} \frac{\partial {\mathscr {E}}_z(z)}{\partial z} - \left[ k_x^2 - \frac{\omega ^2 \varepsilon (z,\omega )}{c^2} - \frac{1}{\varepsilon (z,\omega )} \frac{\partial ^2\varepsilon (z,\omega )}{\partial z} + \left( \frac{1}{\varepsilon (z,\omega )} \frac{\partial \varepsilon (z,\omega )}{\partial z} \right) ^2 \right] {\mathscr {E}}_z(z) = 0, \end{aligned} \end{aligned}$$

where *c* is the speed of light in vacuum. Applying these two differential equations, we can find the wave mode solutions for each region of the Schottky junction.

#### Region A

In this region $\varepsilon (z,\omega ) = \varepsilon _m(\omega )$ is not depending on the *z*-directional space coordinates, and we can identify that

35 $$\begin{aligned} \frac{\partial ^2{\mathscr {E}}_z(z)}{{\partial z}^2} - \kappa _A^2 {\mathscr {E}}_z(z) = 0, \quad \text {where} \quad \kappa _A^2 = \left[ k_x^2 - \frac{\omega ^2 \varepsilon _m(\omega )}{c^2} \right] . \end{aligned}$$

Then we can solve this equation and obtain the solutions

36 $$\begin{aligned} {\mathscr {E}}_z(z) = {\mathscr {E}}_A e^{\kappa _A z}, \quad \text {and} \quad {\mathscr {E}}_x(z) = \frac{i\kappa _A}{k_x} {\mathscr {E}}_A e^{\kappa _A z} \end{aligned}$$

Here, we only considered the ${\text{Re}} [\kappa _A] > 0$ solutions (with $z<0$ values) as we need only the decaying solution on the outside of the interface, and ${\mathscr {E}}_A$ is an unknown real constant.

#### Region B

In this region, $\varepsilon (z,\omega ) = \varepsilon _{hs}$ is a real-valued constant. Thus,

37 $$\begin{aligned} \frac{\partial ^2{\mathscr {E}}_z(z)}{{\partial z}^2} - \kappa _B^2 {\mathscr {E}}_z(z) = 0, \quad \text {where} \quad \kappa _B^2 = \left[ k_x^2 - \frac{\omega ^2 \varepsilon _{hs}}{c^2} \right] . \end{aligned}$$

For $\kappa _B^2 > 0$, we can express the solutions as

38 $$\begin{aligned} {\mathscr {E}}_z(z) = {\mathscr {E}}_{B1} e^{\kappa _B z} + {\mathscr {E}}_{B2} e^{-\kappa _B z} \quad \text {and} \quad {\mathscr {E}}_x(z) = \frac{i \kappa _B}{k_x} \left[ {\mathscr {E}}_{B1} e^{\kappa _B z} - {\mathscr {E}}_{B2} e^{-\kappa _B z} \right] , \end{aligned}$$

where ${\mathscr {E}}_{B1}$ and ${\mathscr {E}}_{B2}$ are unknown constants. However, under the scenario $\kappa _B^2 < 0$, we need to introduce different type of solutions such as

39 $$\begin{aligned} {\mathscr {E}}_z(z) = {\mathscr {E}}_{B1} \cos \left( \sqrt{-\kappa _B^2} z\right) + {\mathscr {E}}_{B2} \sin \left( \sqrt{-\kappa _B^2} z\right) , \quad \text {and} \quad {\mathscr {E}}_x(z) = \frac{i \sqrt{-\kappa _B^2}}{k_x} \left[ -{\mathscr {E}}_{B1} \sin \left( \sqrt{-\kappa _B^2} z\right) + {\mathscr {E}}_{B2} \cos \left( \sqrt{-\kappa _B^2} z\right) \right] . \end{aligned}$$

#### Region C

In this region, charge carrier density varies linearly. Thus, the permittivity function of this region is also dependent on the *z*-directional coordinates. Before further analysis, we should consider the practical aspect of these parameters. Our analysis uses an excitation field frequency $\omega$ in $\sim 10^{15} {{\hbox {s}}^{-1}}$ order. In addition, we model the semiconductor using the parameters of n-type doped gallium arsenide material, and we can identify that its damping factor is in $\sim 10^{12} {{\hbox {s}}^{-1}}$ order. Under these practical conditions, we can assume that $\varsigma _{s} \ll \omega$, and neglect the effects of damping in the semiconductor region. It is important to notice that for $d_1 < z \le d_2$, we can assume that

40 $$\begin{aligned} k_x^2 \ll \left[ \frac{1}{(z-\nu )^2} - \frac{\omega ^2 \varrho }{c^2} (z - \nu )\right] , \quad \text {where} \quad \varrho = - \varepsilon _{hs} \left[ \frac{r\omega _{ps0}^2}{\omega ^2} \right] , \quad \text {and} \quad \nu = - \frac{\varepsilon _{hs}}{\varrho } \left[ 1 + \frac{h\omega _{ps0}^2}{\omega ^2} \right] . \end{aligned}$$

This leads to

41 $$\begin{aligned} \frac{\partial ^2{\mathscr {E}}_z(z)}{{\partial z}^2} + \frac{1}{(z-\nu )} \frac{\partial {\mathscr {E}}_z(z)}{\partial z} - \left[ - \frac{\omega ^2 \varrho }{c^2} (z - \nu ) + \frac{1}{(z-\nu )^2} \right] {\mathscr {E}}_z(z) = 0, \end{aligned}$$

and we can present the solutions as

42 $$\begin{aligned} {\mathscr {E}}_z(\zeta ) = {\mathscr {E}}_{C1} \frac{{\text {Ai}}'(\zeta )}{\zeta } + {\mathscr {E}}_{C2} \frac{{\text {Bi}}'(\zeta )}{\zeta }, \quad \text {and} \quad {\mathscr {E}}_x(z) = \frac{i}{k_x} \left[ \frac{{\mathscr {E}}_{C1}}{\vartheta } \text {Ai}(\zeta ) + \frac{{\mathscr {E}}_{C2}}{\vartheta } \text {Bi}(\zeta ) \right] . \end{aligned}$$

Here, $\zeta = {(z -\nu )}/{\vartheta }$, $\vartheta = \root 3 \of {{c^2}\big /{\varepsilon _{hs} r\omega _{ps0}^2}}$, ${\text {Ai}}'(\zeta )$ is first derivate with respect to $\zeta$ of the first kind of Airy function ${\text {Ai}}(\zeta )$, ${\text {Bi}}'(\zeta )$ is first derivate with respect to $\zeta$ of the second kind of Airy function ${\text {Bi}}(\zeta )$, and ${\mathscr {E}}_{C1}$ and ${\mathscr {E}}_{C2}$ are unknown constants.

#### Region D

Since the dielectric function in this region is a contact respect to the *z*-directional space coordinates, we can observe that

43 $$\begin{aligned} \frac{\partial ^2{\mathscr {E}}_z(z)}{{\partial z}^2} - \kappa _D^2 {\mathscr {E}}_z(z) = 0, \quad \text {and} \quad \kappa _D^2 = \left[ k_x^2 - \frac{\omega ^2 \varepsilon _s(\omega )}{c^2} \right] . \end{aligned}$$

Same as the region A, we can present the solution as

44 $$\begin{aligned} {\mathscr {E}}_z(z) = {\mathscr {E}}_D e^{-\kappa _D z} \quad \text {and} \quad {\mathscr {E}}_x(z) = -\frac{i\kappa _D}{k_x} {\mathscr {E}}_D e^{-\kappa _D z} \end{aligned}$$

Here, we only considered the ${\text{Re}} [\kappa _D] > 0$ solutions (with $z > d_2$ values) as we need only the decaying solution on the outside of the interface, and ${\mathscr {E}}_D$ is an unknown real constant.

### Dispersion relation of SPP modes at the Schottky junction

The dispersion relation for possible SPP modes relates the excitation light’s angular frequency to the SPP mode’s in-plane wavenumber magnitude. The dispersion relation can be found by applying the self-consistent boundary conditions required by Maxwell’s equations^36^

45 $$\begin{aligned} \varepsilon _{\text {R}1} {\mathscr {E}}_{z,\text {R}1}(z) = \varepsilon _{\text {R}2} {\mathscr {E}}_{z,\text {R}2}(z), \quad \text {and} \quad {\mathscr {E}}_{x,\text {R}1}(z) = {\mathscr {E}}_{x,\text {R}2}(z), \end{aligned}$$

where $\text {R}1,\text {R}2 \in \{\text {A, B, C, D}\}$ at $z=0$, $z=d_1$, and $z=d_2$ to ensure the continuity of the SPP electromagnetic field. Using the previously identified wave modes for each region in the Schottky junction, we can derive six linear simultaneous equations for these boundary conditions, and represent them in a matrix equation

46 $$\begin{aligned} {M}_{6\times 6}(\omega , k_x) {{\mathscr {E}}}_{6\times 1} = 0. \end{aligned}$$

Here, ${M}_{6\times 6}(\omega , k_x)$ is the matrix of coefficients, and it can be represented as

47 $$\begin{aligned} {M}_{6\times 6}(\omega , k_x) = \begin{bmatrix} \varepsilon _m(\omega ) &{} -\varepsilon _{hs} &{} -\varepsilon _{hs} &{} 0 &{} 0 &{} 0 \\ \kappa _A &{} -\kappa _B &{} \kappa _B &{} 0 &{} 0 &{} 0 \\ 0 &{} e^{\kappa _B d_1} &{} e^{-\kappa _B d_1} &{} - \frac{{\text {Ai}}'(\zeta _{d_1})}{\zeta _{d_1}} &{} - \frac{{\text {Bi}}'(\zeta _{d_1})}{\zeta _{d_1}} &{} 0 \\ 0 &{} \kappa _B e^{\kappa _B d_1} &{} - \kappa _Be^{-\kappa _B d_1} &{} -\frac{{\text {Ai}}(\zeta _{d_1})}{\vartheta } &{} -\frac{{\text {Bi}}(\zeta _{d_1})}{\vartheta } &{} 0 \\ 0 &{} 0 &{} 0 &{} \frac{{\text {Ai}}'(\zeta _{d_2})}{\zeta _{d_2}} &{} \frac{{\text {Bi}}'(\zeta _{d_2})}{\zeta _{d_2}} &{} -e^{-\kappa _D d_2} \\ 0 &{} 0 &{} 0 &{} \frac{{\text {Ai}}(\zeta _{d_2})}{\vartheta } &{} \frac{{\text {Bi}}(\zeta _{d_2})}{\vartheta } &{} \kappa _D e^{-\kappa _D d_2} \\ \end{bmatrix}, \end{aligned}$$

for $\kappa _B^2 > 0$. However, under the $\kappa _B^2 < 0$ condition, we can find that

48 $$\begin{aligned} {M}_{6\times 6}(\omega , k_x) = \begin{bmatrix} \varepsilon _m(\omega ) &{} -\varepsilon _{hs} &{} 0 &{} 0 &{} 0 &{} 0 \\ \kappa _A &{} 0 &{} -\kappa _{B'} &{} 0 &{} 0 &{} 0 \\ 0 &{} \cos (\kappa _{B'} d_1) &{} \sin (\kappa _{B'} d_1) &{} - \frac{{\text {Ai}}'(\zeta _{d_1})}{\zeta _{d_1}} &{} - \frac{{\text {Bi}}'(\zeta _{d_1})}{\zeta _{d_1}} &{} 0 \\ 0 &{} -\kappa _{B'}\sin (\kappa _{B'} d_1) &{} \kappa _{B'}\cos (\kappa _{B'} d_1) &{} -\frac{{\text {Ai}}(\zeta _{d_1})}{\vartheta } &{} -\frac{{\text {Bi}}(\zeta _{d_1})}{\vartheta } &{} 0 \\ 0 &{} 0 &{} 0 &{} \frac{{\text {Ai}}'(\zeta _{d_2})}{\zeta _{d_2}} &{} \frac{{\text {Bi}}'(\zeta _{d_2})}{\zeta _{d_2}} &{} -e^{-\kappa _D d_2} \\ 0 &{} 0 &{} 0 &{} \frac{{\text {Ai}}(\zeta _{d_2})}{\vartheta } &{} \frac{{\text {Bi}}(\zeta _{d_2})}{\vartheta } &{} \kappa _D e^{-\kappa _D d_2} \\ \end{bmatrix}, \end{aligned}$$

where $\kappa _{B'} = \sqrt{-\kappa _B^2}$, and ${{\mathscr {E}}}_{6\times 1} = [{\mathscr {E}}_{A} , {\mathscr {E}}_{B1} , {\mathscr {E}}_{B2} , {\mathscr {E}}_{C1} , {\mathscr {E}}_{C2} , {\mathscr {E}}_{D} ]^{{\textsf{T}}}$. A nontrivial solution to ${{\mathscr {E}}}_{6\times 1}$ can be obtained by ensuring that the determinant of the coefficient matrix ${M}_{6\times 6}(\omega , k_x)$ is equal to zero

49 $$\begin{aligned} \det ({M}_{6\times 6}(\omega , k_x) ) = 0, \end{aligned}$$

and this leads to a secular equation relating $\omega$ and $k_x$. This is the dispersion relation for the possible SPP modes in a Schottky junction.

## Results and discussion

This section presents the numerical results we received for the characteristics of SPP modes in dressed Schottky junction-based waveguides. In this analysis, we consider silver (Ag) metallic material and n-doped gallium arsenide (GaAs) semiconductor material. To ensure the validity of the free electron model, a plasmonic metal with minimal natural damping effects needs to be chosen. Ag was selected as it has the lowest natural damping factor among popular plasmonic materials^34^. Additionally, Ag is a better candidate for dressed SPP modes with long propagation lengths, while other properties remain unaffected^22^. In prior studies on Schottky junctions, n-doped silicon (Si) or n-doped GaAs were used as the semiconductor material^16,17^. The selection of the semiconductor material for the Schottky junction does not impact the research outcomes since the main focus is on reducing propagation losses in the metallic region. However, selecting a semiconductor with a higher plasma frequency can provide a higher frequency range for operating SPP modes. GaAs was selected over doped Si as it has a lower effective electron mass, resulting in a higher plasma frequency. Unless specified otherwise, the following empirical material parameters are used in the numerical calculations^37,38^: the metal plasma frequency $\omega _{pm} = 1.352 \times {10^{16}} \, {{\hbox {rads}}^{-1}}$, natural damping factor $\gamma _0 = {0.2} \, \hbox {eV}$, the high-frequency permittivity $\varepsilon _{hm} = 5$, and energy $\epsilon _F = {5.5} \, \hbox {eV}$ for Ag. The effective mass *m* is $1.0m_e$, and $0.067m_e$ for Ag and GaAs respectively. Here, $m_e$ is the mass of an electron. Furthermore, we assume static dielectric constant $\varepsilon _{hs} = 12.3$, and bulk carrier density $n_s = 2 \times {10^{24}} \, {{\hbox {m}}^{-3}}$ for GaAs. According to Newman’s research^39^, this bulk carrier density is achievable for n-doped GaAs. With these empirical data, we can evaluate the bulk plasma frequency for the semiconductor region and obtain $\omega _{ps0} = 8.789 \times {10^{13}} \, {{\hbox {rads}}^{-1}}$. In addition, we assume that the dressing field frequency $\Omega = 1 \times {10^{14}} \, {{\hbox {rads}}^{-1}}$, and the reference dressing field intensity $I_0 = {3} \, {{\hbox {kWcm}}^{-2}}$. The full mathematica code for the numerical calculations is available under the Supplementary files.

### Dispersion relation with lossy metal

The previous literature^17,19^ assumed that the dielectric function of the metal is a real-valued function for all frequency ranges. Under the ${\tilde{\omega }} < w$ conditions, it is not fair to use this assumption for dispersion relation calculations. Here, we introduced the normalized frequencies $w = {\omega _{pm}}/{\omega _{ps0}}$, and ${\tilde{\omega }} = {\omega }/{\omega _{ps0}}$. For our calculations, we can identify that for all the SPP operating frequency range ${\tilde{\omega }} < w$. Thus, we can not neglect the effects of the imaginary part of the dielectric function under our calculations.

**Figure 2:**
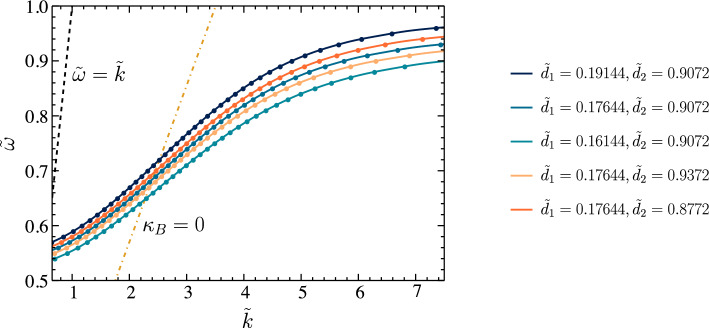
Dispersion relation (${\tilde{k}}-{\tilde{\omega }}$) for different values of $d_1$ and $d_2$. Here, ${\tilde{k}} = {{\text{Re}} [k_x]}/{\Delta }$, and $\Delta = {c}/{\omega _{sp0}}$. Furthermore, $\tilde{d}_1 = {d_1}/{\Delta }$, and $\tilde{d}_2 = {d_1}/{\Delta }$.

**Figure 3:**
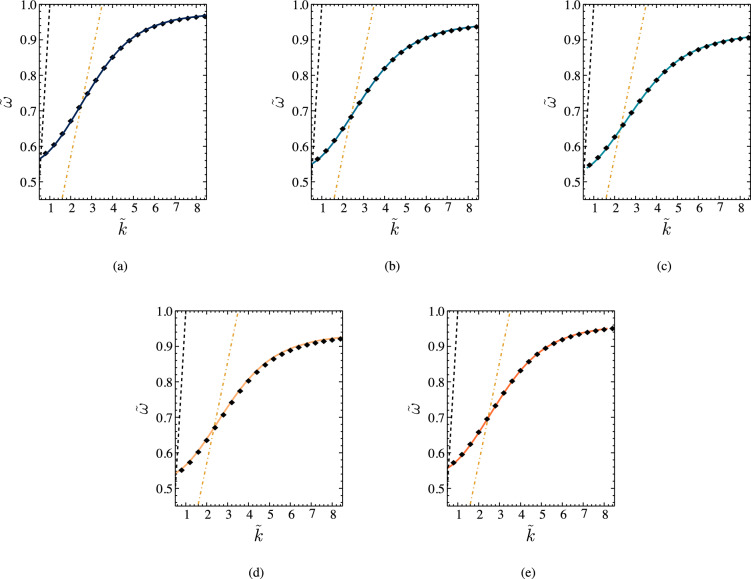
Comparison of the dispersion relation under the lossy metal condition and lossless metal condition for different values of $d_1$ and $d_2$. The black dots represent the dispersion relationship for lossless metal conditions. Here, ${\tilde{k}} = {{\text{Re}} [k_x]}/{\Delta }$, and $\Delta = {c}/{\omega _{sp0}}$. Furthermore, $\tilde{d}_1 = {d_1}/{\Delta }$, and $\tilde{d}_2 = {d_2}/{\Delta }$.

By solving the expression given in Eq. (49) under the assumption that the metal is a lossy medium, we can get dispersion relation for different ${\tilde{d}}_1$ and ${\tilde{d}}_2$ values as given in Fig. 2. We must note that we can identify two separate SPP modes in a Schottky junction^17^. Our analysis, however, focuses on the high-frequency SPP mode, similar to the SPP dispersion of a bi-metal system. Figure 3a–e present the comparison of dispersion relation under lossy metal condition and lossless metal condition for different ${\tilde{d}}_1$ and ${\tilde{d}}_2$ values. It appears that the changes in the damping factor are insufficient to affect the dispersion relation, and the dressing field can not alter the dispersion relation of the natural Schottky junction-based SPP modes. In terms of the dispersion relation, the lossy metal results are very similar to the lossless metal results^17^. Each SPP mode has a maximum achievable frequency defined by $d_1$ and $d_2$. This explains how the SPP properties may be manipulated by adjusting the dimension of the space charge region. Nevertheless, we must not ignore one important result of lossy metal calculations. For each ${\tilde{k}}$ value in the above relationship, we find that the imaginary part of it is not zero. The imaginary part is responsible for the energy losses that occur within the metal region. This results in a finite propagation length of the considered SPP modes due to the energy losses. This scenario can be observed in real materials, and these effects should be considered in the context of a comprehensive Schottky junction-based SPP waveguide analysis.

### SPP propagation length analysis

The finite propagation length associated with SPP modes is a significant barrier to advancing plasmonic devices. Long-distance electromagnetic wave transfers are not achievable in plasmonic devices due to this reason. Therefore, researchers have been focusing on finding new methods to improve the SPP propagation length in recent years. One promising solution is using Floquet engineering methods, which can increase the propagation length in these systems^22^. By using an external electromagnetic field, the wave function of metal electrons can be changed, reducing electron impurity scattering and the damping factor for the conduction electrons in the metal region can also be reduced.

**Figure 4:**
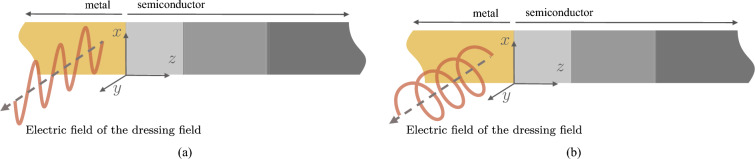
The metal region is illuminated by (**a**) a linearly polarized dressing field, and (**b**) a circularly polarized dressing field. The applied field is perpendicular to the *xz*-plane. The linearly polarized field consists an *x*-polarized electric field.

Now, we consider the effect of the dressing field on the Schottky junction-based plasmonic waveguides. Especially the effects induced on the imaginary part of the SPP wavenumber. Here, we analyze the impact of two different types of dressing fields: (i) linearly polarized dressing field, and (ii) circularly polarized dressing field. We assume that these fields propagate perpendicularly to the *xz*-plane, as illustrated in Fig. 4. Moreover, we model the linearly polarized radiation with an *x*-polarized electric field. First, we can express the normalized damping factor for the metal electrons under the linear-polarized dressing field^22,31^ (see the Supplementary Information, Section 3)

50 $$\begin{aligned} {\tilde{\gamma }}_{\text {linear}} = \frac{1}{16\pi ^2} \int _{0}^{\pi } \int _{0}^{2\pi } \sin \varphi \left[ \int _{0}^{\pi } \int _{0}^{2\pi } \sin \varphi ' J_0^2 \left( \Lambda \left[ \sin \vartheta \sin \varphi - \sin \varphi '\sin \vartheta '\right] \right) {\textrm{d}}\vartheta ' {\textrm{d}}\varphi ' \right] {\textrm{d}}\vartheta {\textrm{d}}\varphi . \end{aligned}$$

Here, $\Lambda = {e\xi k_F}/{m\Omega ^2}$, $k_F = \sqrt{2m\epsilon _F}$, $\xi$ is the amplitude of the electric field of the dressing field, and $J_l(\cdot )$ are Bessel functions of the first kind with *l*-th integer order. Under the circular-polarized dressing field, the normalized damping factor for the dressed metal electrons can be expressed as follows:

51 $$\begin{aligned} {\tilde{\gamma }}_{\text {circular}} = \frac{1}{4} \int _{0}^{\pi } \sin \varphi \sum _{m=-\infty }^{\infty } \left[ \int _{0}^{\pi } \sin \varphi ' J_m^2\left( \Lambda \sin (\varphi )\right) J_m^2\left( \Lambda \sin (\varphi ')\right) {\textrm{d}}\varphi ' \right] {\textrm{d}}\varphi . \end{aligned}$$

In our derivations, we characterized the stationary scattering potential based on impurities as a collection of randomly distributed impurity potentials. To model this single scattering potential, we utilized the Gaussian model framework with the white noise approximation^26,40,41^. It is important to highlight that this theoretical framework depends on both the type of impurities present and the electron density of the metal. Readers interested in the details of the impurity model can find them in the Supplementary Information file, Section 2.

Before analyzing the numerical results of possible propagation length improvements in SPP modes, we define the SPP propagation length on the *x*-direction as follows

52 $$\begin{aligned} L_{\text {spp}} = \frac{1}{2\text {Im}[k_x]}. \end{aligned}$$

**Figure 5:**
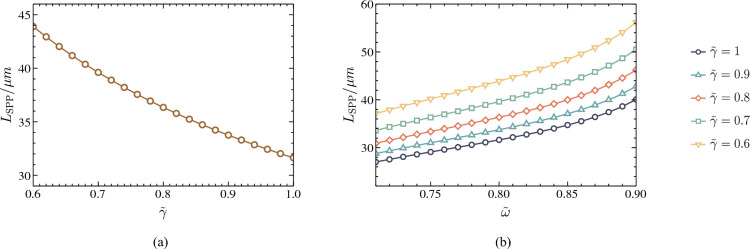
(**a**) The SPP propagation length $L_{\text {spp}}$ against the normalized damping factor ${\tilde{\gamma }}$ when ${\tilde{\omega }} =0.8$. (**b**) The SPP propagation length $L_{\text {spp}}$ against the normalized frequency ${\tilde{\omega }}$ for different damping factor values. For all the scenarios, we assumed the $\tilde{d}_1 = 0.17644$ and $\tilde{d}_2 = 0.9072$. Moreover, $L_{0}$ is the natural propagation length of the Schottky SPP mode, and $I_0$ is the reference dressing field intensity.

The first step is to change the metal damping factor to analyze the propagation length changes. For an example, we illustrated the Schottky SPP propagation length against the damping factor scaling parameter for frequency ${\tilde{\omega }} = 0.8$ in Fig. 5a. Here, we assumed that $\tilde{d}_1 = 0.17644$ and $\tilde{d}_2 = 0.9072$. In this figure, we can identify that the propagation length can be increased by reducing the ${\tilde{\gamma }}$ value for a given frequency. Especially if we can decrease the metal region’s damping factor, we can achieve improved propagation lengths in Schottky junction-based SPP modes.

**Figure 6:**
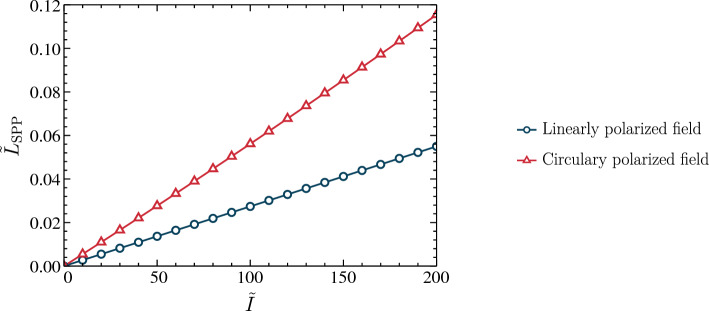
The normalized SPP propagation length different factor ${\tilde{L}}_{\text {spp}} = {(L_{\text {spp}} - L_{0})}/{L_{0}}$ against the normalized dressing field intensity ${\tilde{I}} = {I}/{I_0}$ when ${\tilde{\omega }} =0.8$.

Next, we calculate the finite SPP propagation length against a frequency range for different damping factors given that $\tilde{d}_1 = 0.17644$ and $\tilde{d}_2 = 0.9072$. The result is depicted in Fig. 5b. As the frequency decreases, the propagation length decreases as well. The reason for this is that the imaginary part of the dielectric function increases as the frequency decreases. In addition, we can identify that throughout the whole frequency range, we can achieve high propagation length by decreasing the damping factor.

In a previous study, we comprehensively analyzed the effects of external dressing field on ${\tilde{\gamma }}_{\text {linear}}$ and ${\tilde{\gamma }}_{\text {circular}}$ parameters and showed that increasing the dressing field intensity, we can achieve a lower damping factor^22,31^. Using this insight, we present the relationship between the normalized Schottky SPP propagation length difference factor ${\tilde{L}}_{\text {spp}} = {(L_{\text {spp}} - L_{0})}/{L_{0}}$ and normalized dressing field’s intensity ${\tilde{I}} = {I}/{I_0}$ for ${\tilde{\omega }} = 0.8$ in Fig. 6. Here, $L_{0}$ is the natural propagation length of the Schottky junction-based SPP mode for ${\tilde{\omega }} = 0.55$, and $I_0$ is the reference dressing field intensity. Examining these results, we can identify that the normalized propagating length exhibit a positive valued normalized propagation length difference factor, and it can be enhanced by increasing the dressing field intensity. This allows us to improve the propagation length of SPP modes using an external dressing field.

As shown by the numerical analysis of the dressing field effect on the dispersion relation, there is no significant change. Conversely, we observe that a dressing field irradiated onto the Schottky junction significantly increases the propagation length of SPPs. To evaluate the effectiveness of our proposed method, we need to compare it against the previously proposed methods for improving SPP modes based on Schottky junctions. There have been previous studies^17,23^ that recommend applying an externally biased voltage across the junction below the breakdown voltage of the semiconductor to improve the SPP propagation length. Their method, however, involves changing the characteristics of the Schottky junction’s space charge region. A limitation of this method is that it also changes the dispersion relation of the possible SPP modes, as illustrated in Fig. 2. The Floquet engineering-based technique, in contrast, allows us to tailor the propagation length of any given SPP mode without modifying its SPP wavelength, decay lengths, or dispersion relation extensively^22^. As a result, our method is more suitable for high-sensitivity nanoplasmonic devices and circuitry. In addition, it is essential to note that the propagation length of the SPP can be modified differently by each polarization type. According to outcomes in Fig. 6, circular-polarized dressing fields are capable of increasing propagation lengths in comparison with linear-polarized dressing fields. Based on the results, circularly polarized radiation appears to be capable of improving propagation lengths more significantly. Furthermore, by looking at the expressions in Eqs. (50) and (51), we can identify that the damping factor also depends on the dressing field’s frequency. Consequently, we can increase the SPP propagation length by lowering the dressing field’s frequency within a certain range. As a result of these observations, it is evident that the modification in SPP propagation length at dressed Schottky junctions is determined by the intensity of the dressing field and the polarization type and frequency. We believe this is an additional advantage of our proposed method compared with the previous studies. Subsequently, we can put our findings into practice in developing nanoscale information processing techniques and plasmonic sensors.

The strong subwavelength localization of SPP modes in plasmonic nanostructures is a driving force behind the recent development of SPP-based information carriers^24^. The intrinsic losses of SPPs, which restrict the propagation length of the SPP mode, are the primary concern when it comes to achieving plasmonic circuitry. When the propagation length of SPP is short, it results in higher thresholds of plasmonic nanolasers^42,43^, low efficiency of SPP waveguiding and focusing^22,44,45^, and lower Purcell factor of quantum nanoplasmonics resonators^46,47^. Several experimental and theoretical methods have been proposed to attain high-efficiency plasmon circuitry, with the Schottky junction-based devices being one of the most promising solutions^16,48,49^. The Schottky junction enables the introduction of minority carriers into the junction by altering the bias voltage, as discussed in previous literature^16,17^, which amplifies the SPP mode and provides for higher propagation lengths. However, a potential drawback is that it can alter other SPP characteristics and may not be ideal for applications such as sensing that require very low ambiguity on the characteristics of SPP while applying improvements^50^. We believe that our dressed SPP method could effectively enhance the efficiency of plasmonic sensor applications^50–52^, as it offers an improved propagation length while keeping other characteristics unchanged. Furthermore, the dependence of the propagation length on the frequency and polarization of the dressing field provides an opportunity to employ it in chip-scale wireless communication^53,54^. For instance, the data signal can be frequency modulated into the dressing field and transmitted from the transmitter. The received signal can be then applied to a Schottky junction-based waveguide at the receiver. Since the strength of the existing SPP modes is contingent on the frequency of the dressing field, the SPP mode’s intensity changes in response to the data signal. This observation allows for the use of dressed Schottky junction-based waveguides to construct next-generation frequency demodulators for chip-scale wireless communications.

Irradiating a metallic system with a dressing field increases SPP propagation length because the probability of electron scattering changes due to the dressing field. The phenomenon of electron scattering occurs when moving electrons are reflected in another direction. In consequence, electron scattering causes electrons to lose their kinetic momentum and results in finite SPP propagation lengths. The scattering of electrons in a metallic system occurs through several mechanisms, including both elastic scattering processes caused by impurities, and inelastic scattering processes caused by phonons. As mentioned in Rudner et al.^55^, by properly selecting the frequency of the dressing field and considering the system under short-time scales, we can ensure that our Floquet system is under stable low-temperature conditions. Given the low-temperature conditions, it is reasonable to assume that elastic scattering holds greater significance^41,56,57^. Therefore, the damping effects in electron transport under our investigation are mainly due to electron scattering by disorder impurities. The overlap between the wave functions of an incident electron and a scattered electron can be used to illustrate the probability of scattering, as outlined in the fundamental Floquet–Fermi golden rule. This rule is a generalization of Fermi’s golden rule^58,59^ that pertains to quantum systems subjected to periodic driving. It enables the computation of the transition rate between Floquet states. A detailed derivation and discussion of the Floquet–Fermi’s golden rule can be found in several recent literatures^22,26,60–62^. Since wave function terms are dependent on the dressing field intensity, higher field intensities can reduce the stationary overlap of wave functions and, thus, the scattering probability. This results in a reduction in the scattering probability at high fields, and low-temperature steady states, causing the SPP propagation length to be enhanced as a result. An important point to remember is that a generic interacting Floquet system absorbs photons from the radiation and tends to produce heat. To describe a general dressed system completely, the interaction between photons must be taken into account. There is a considerable amount of complexity associated with these interactions. For overcoming the challenge of heating and achieving non-equilibrium steady-state, various strategies are available^55^. It is possible to achieve steady particle distribution functions in driven isolated quantum systems by working in regimes where heating rates are strongly suppressed. Accordingly, we carefully selected the dressing frequency and time scales in our analysis.

It is essential to acknowledge that, similar to any scientific technique or approach, the ability to enhance the carrier transport properties of materials through Floquet engineering via radiation is subject to unique limitations and considerations regarding its applicability. Floquet engineering involves the manipulation and control of quantum systems under periodic driving, which is governed by several parameters, including driving frequency, driving intensity, system Hamiltonian, and others^22,63^. The appropriate selection and tuning of these parameters enable the exploration of various phenomena, the realization of novel states of matter, and the achievement of desired quantum functionalities. In Floquet engineering, increasing the dressing frequencies leads to a wider energy gap between Floquet levels, resulting in reduced band overlapping and intraband absorptions^26,63^. Conversely, lower frequencies induce significant changes in the wavefunction and minimize the damping factor. Hence, careful consideration should be given to the choice of dressing field frequency to attain optimal results. The behavior of Floquet modes is primarily influenced by the intensity of the dressing field^22,26^. Higher intensities induce significant changes in the wavefunction, reducing the damping factor and improving carrier transportation. However, excessively high-intensity fields can cause material fluidization, necessitating the careful selection of intensity levels to preserve the integrity of the samples^57^. The selection of an appropriate intensity level is crucial in addressing carrier collisions, energy broadening, and dephasing, taking into account the material properties and experimental setups. The optimal intensity range varies depending on the specific material and system under investigation. Furthermore, the system Hamiltonian plays a crucial role in dressed quantum systems. The applicability of the free electron model used in this study may not be universal, as complex energy band structures present challenges in determining suitable dressing field parameters. The presence of external fields can further modify the system Hamiltonian, limiting the feasibility of achieving desired improvements^62^. To make informed decisions in Floquet engineering research, it is crucial to understand the limitations and compatibility of the approach. While not all materials benefit from Floquet engineering enhancements, those with low energy band broadening, such as semiconductor-based low dimensional electron gas and high-mobility metals, have been favored in many literature studies^22,26,57,62,64^. These considerations lead us to select Ag as the metallic material in our system, along with other appropriate dressing field parameters.

Schottky junctions can have their space charge region’s width altered by applying an additional voltage, allowing for active manipulation of Schottky SPP characteristics, which can be used in many nanoplasmonic devices^17,24,65^. These devices also have several attractive features, including low cost, ease of fabrication, and integration with semiconductor electronics and sensors^16,17,23^. However, these junctions have limited field enhancements and propagation distances due to intrinsic losses in the interface materials. To improve SPP propagation length in Schottky interfaces, Floquet engineering methods can be used without changing any other SPP property. One constraint of the Floquet engineering technique that has been presented is that significant enhancements in propagation length can only be achieved by limiting the frequency range of the SPPs to the high terahertz range. Nonetheless, there exist several applications utilizing plasmonic waveguides within this frequency range which are associated with Schottky junctions^52,66^. These insights can be used to model SPP propagation in Schottky waveguides, which are simple, energy-efficient, and easier to integrate into nanoplasmonic devices.

## Conclusions

The behavior of SPP modes at a Schottky junction under un-driven and driven conditions was presented. A comprehensive analytical study on the dressed metal dielectric function was also presented, using the linear response theory and Floquet theory to derive the dressed metal susceptibility function. It was identified that the dressing field only influences the metal electron’s damping factor. Using Maxwell’s equations and free electron model assumptions, the dispersion relation of SPP modes was expressed, with consideration for the effects of intrinsic energy losses associated with the metallic region. The dispersion relation provided similar results as the lossless assumption-based results, but energy losses were demonstrated to be due to the electron scattering caused by impurities, which was overlooked in previous studies. Analytical expressions for the damping factor of the metallic system under two types of polarized dressing fields were introduced. The potential of increasing SPP propagation length was demonstrated through a detailed numerical analysis with empirical system and material parameters. The propagation length changes depend on the dressing field’s intensity, the polarization type, and the frequency. The method is unique in its ability to improve SPP propagation length while retaining other characteristics of SPP modes. These changes are primarily due to the dressing field modifying the free electron wave function in the metal, which is responsible for the reduction in impurity-based electron scattering. The external dressing field can be used to reduce the damping effects in a Schottky junction and improve the propagation length of related SPP modes to achieve next-generation plasmonic waveguides. The derivations and results provide the much-needed theoretical background for understanding and designing advanced Schottky junction-based waveguides and will guide the development and optimization of a wide spectrum of SPP waveguiding-based nanoplasmonic devices.

## Supplementary Information

Supplementary Information.

## Data Availability

All data generated or analyzed during this study are included in this published article and its supplementary files.
