# Supplementary material for: A Floquet engineering approach to optimize Schottky junction-based surface plasmonic waveguides
Source: Sci Rep. 2023 Jul 2;13:10692. doi: 10.1038/s41598-023-37801-x (PMC10315399; doi:10.1038/s41598-023-37801-x)
Supplement: Supplementary file 1 — Supplementary Information. [file 41598_2023_37801_MOESM1_ESM.pdf]

A Floquet engineering approach to optimize Schottky junction-based surface plasmonic waveguides: Supplementary information

Kosala Herath[1], Sarath D. Gunapala[2], and Malin Premaratne[1,*]

[1]Advanced Computing and Simulation Laboratory (AχL), Department of Electrical and Computer Systems Engineering, Monash University, Clayton, Victoria 3800, Australia.

[2]Jet Propulsion Laboratory, California Institute of Technology, Pasadena, California 91109, USA.

*malin.premaratne@monash.edu

1 Dielectric function of dressed metals

In this section, we are trying to derive an expression for the optical conductivity of a dressed metallic system. We consider a thin metallic film under a high-intensity external field. We extend the equilibrium linear response theory to a strongly driven system out of equilibrium, which is probed by a weak external potential. Furthermore, we derive an expression for the current response that is not only depend on the frequency of the probe but the frequency of the drive. Finally, we present an analytical expression for the susceptibility function and dielectric function of the dressed metal.

1.1 Kubo formula for conductivity

The linear response theory states that the response to a weak external perturbation is proportional to the perturbation, and therefore all we need to understand is the proportionality constant. The correction functions which describe the linear response to the perturbation are named as *Kubo formulas*. Since we are trying to find the susceptibility function from the averaged current density, this particular analysis is on the electrical conductivity that linearly corrects the current response to a perturbing electric field.

As the first step, we derive the generalized Kubo formula under the linear response theory. Consider a system defined by the Hamiltonian

$$\hat{H}(t) = \hat{H}_0 + \mathcal{V}(t), \tag{S1}$$

where $\hat{H}_0$ is the Hamiltonian for the system without the time-dependent perturbation $\mathcal{V}(t)$ in the thermodynamic equilibrium. Using basic postulates of statistical mechanics, the thermal expectation value of any quantum operator $\hat{\mathcal{O}}$ which is not explicitly time-dependent observable, is without the perturbation $\mathcal{V}(t)$ can be given by [1]

$$\left\langle \hat{\mathcal{O}} \right\rangle_0 = \mathrm{tr}\left[\hat{\rho}_0 \hat{\mathcal{O}}\right], \tag{S2}$$

with the density operator for a grand canonical ensemble

$$\hat{\rho}_0 = \frac{\exp\left(-\mathcal{B}\hat{\mathcal{H}}_0\right)}{\mathrm{tr}\left[\exp\left(-\mathcal{B}\hat{\mathcal{H}}_0\right)\right]}. \tag{S3}$$

The Hamiltonian of the grand canonical ensemble can be expressed by

$$\hat{\mathcal{H}}_0 = \hat{H}_0 - \mu \hat{N}, \tag{S4}$$

where μ is the chemical potential, $\hat{N}$ is the particle number operator, and $\mathcal{B} = 1/k_B T$ is the inverse temperature parameter. The Boltzmann constant, and the absolute temperature is represented by k_B, and T respectively.

Once the perturbation is turned on, a new equilibrium can be established and the density operator (and its matrix elements) will be modified. Under a nonzero time-dependent perturbation, the thermal expectation value of quantum operator $\hat{\mathcal{O}}$ is the trace over time-dependent density operator and observable operator

$$\left\langle \hat{\mathcal{O}} \right\rangle = \mathrm{tr}\left[\hat{\rho}(t) \hat{\mathcal{O}}\right], \tag{S5}$$

which is a time dependent value. Using *Liouville-von Neumann* equation [1], we can identify the time evolution of the density operator as

$$i\hbar \frac{\partial \hat{\rho}(t)}{\partial t} = [\hat{\mathcal{H}}(t), \hat{\rho}(t)], \tag{S6}$$

where

$$\hat{\mathcal{H}}(t) = \hat{H}(t) - \mu\hat{N}. \tag{S7}$$

Then, we can write the time-dependent density matrix as

$$\hat{\rho}(t) = \hat{\rho}_0 + \hat{\rho}'(t), \tag{S8}$$

and substituting back into the equation (S6), we can get

$$i\hbar\frac{\partial\hat{\rho}(t)}{\partial t} = [\hat{\mathcal{H}}_0 + \mathcal{V}(t), \hat{\rho}_0 + \hat{\rho}'(t)]. \tag{S9}$$

Then, this can be expanded as

$$i\hbar\frac{\partial(\hat{\rho}_0 + \hat{\rho}'(t))}{\partial t} = [\hat{\mathcal{H}}_0, \hat{\rho}_0] + [\hat{\mathcal{H}}_0, \hat{\rho}'(t)] + [\mathcal{V}(t), \hat{\rho}_0] + [\mathcal{V}(t), \hat{\rho}'(t)]. \tag{S10}$$

Since $\hat{\rho}_0$ commute with $\hat{\mathcal{H}}_0$, the first term of the right-hand side will be equal to zero. Furthermore, last the term can be neglect as weak perturbation and considering only linear order terms. Then, we can approximate the time evaluation of the density operator as

$$i\hbar\frac{\partial\hat{\rho}'(t)}{\partial t} \approx [\hat{\mathcal{H}}_0, \hat{\rho}'(t)] + [\mathcal{V}(t), \hat{\rho}_0]. \tag{S11}$$

Next, we can switch into the Dirac picture and present the density operator as

$$\hat{\rho}_D(t) = \hat{\rho}_0 + \hat{\rho}'_D(t), \tag{S12}$$

where

$$\hat{\rho}'_D(t) = \exp\left(\frac{i\hat{\mathcal{H}}_0 t}{\hbar}\right)\hat{\rho}'(t)\exp\left(\frac{-i\hat{\mathcal{H}}_0 t}{\hbar}\right). \tag{S13}$$

Then, we can find that

$$i\hbar\frac{\partial\hat{\rho}_D(t)}{\partial t} = [\hat{\rho}'_D(t), \hat{\mathcal{H}}_0] + \exp\left(\frac{i\hat{\mathcal{H}}_0 t}{\hbar}\right)\left\{i\hbar\frac{\partial\hat{\rho}'(t)}{\partial t}\right\}\exp\left(\frac{-i\hat{\mathcal{H}}_0 t}{\hbar}\right). \tag{S14}$$

Take the Dirac picture representation of the equation (S11), and we can identify that

$$\exp\left(\frac{i\hat{\mathcal{H}}_0 t}{\hbar}\right)\left\{i\hbar\frac{\partial\hat{\rho}'(t)}{\partial t}\right\}\exp\left(\frac{-i\hat{\mathcal{H}}_0 t}{\hbar}\right) = [\hat{\mathcal{H}}_0, \hat{\rho}'_D(t)] + [\mathcal{V}_D(t), \hat{\rho}_0]. \tag{S15}$$

Substituting this back into the equation (S14), we can get

$$i\hbar\frac{\partial\hat{\rho}_D(t)}{\partial t} = [\hat{\rho}'_D(t), \hat{\mathcal{H}}_0] + [\hat{\mathcal{H}}_0, \hat{\rho}'_D(t)] + [\mathcal{V}_D(t), \hat{\rho}_0] = [\mathcal{V}_D(t), \hat{\rho}_0] \tag{S16}$$

Assuming the perturbation is switched on at a certain time t_0, and considering a long timescale ($t_0 \to -\infty$), we can identify that

$$\hat{\rho}'_D(t) = -\frac{i}{\hbar}\int_{-\infty}^{t}[\mathcal{V}_D(t'), \hat{\rho}_0]\mathrm{d}t', \tag{S17}$$

and

$$\hat{\rho}_D(t) - \hat{\rho}_0 = -\frac{i}{\hbar}\int_{-\infty}^{t}[\mathcal{V}_D(t'), \hat{\rho}_0]\mathrm{d}t'. \tag{S18}$$

Then, switch back to the Schrödinger's picture

$$\hat{\rho}(t) = \hat{\rho}_0 - \frac{i}{\hbar}\int_{-\infty}^{t} \exp\left(\frac{-i\hat{\mathcal{H}}_0 t}{\hbar}\right)[V_D(t'), \hat{\rho}_0]\exp\left(\frac{i\hat{\mathcal{H}}_0 t}{\hbar}\right)\mathrm{d}t'. \tag{S19}$$

Using this derived expression, one can analyze the time evolution of the thermal averaged value of physical observable $\hat{\mathcal{O}}$. Acting operator $\hat{\mathcal{O}}$ on the left side of the equation (S19), and getting trace over both sides leads to

$$\mathrm{tr}\left[\hat{\rho}(t)\hat{\mathcal{O}}\right] = \mathrm{tr}\left[\hat{\rho}_0\hat{\mathcal{O}}\right] - \frac{i}{\hbar}\int_{-\infty}^{t} \mathrm{tr}\left[\exp\left(\frac{-i\hat{\mathcal{H}}_0 t}{\hbar}\right)[\mathcal{V}_D(t'), \hat{\rho}_0]\exp\left(\frac{i\hat{\mathcal{H}}_0 t}{\hbar}\right)\hat{\mathcal{O}}\right]\mathrm{d}t'. \tag{S20}$$

After applying the trace operator's invariant property under cyclic permutations, we can observe that

$$\mathrm{tr}\left[\hat{\rho}(t)\hat{\mathcal{O}}\right] = \mathrm{tr}\left[\hat{\rho}_0\hat{\mathcal{O}}\right] - \frac{i}{\hbar}\int_{-\infty}^{t} \mathrm{tr}\left[[\mathcal{V}_D(t'), \hat{\rho}_0]\exp\left(\frac{i\hat{\mathcal{H}}_0 t}{\hbar}\right)\hat{\mathcal{O}}\exp\left(\frac{-i\hat{\mathcal{H}}_0 t}{\hbar}\right)\right]\mathrm{d}t', \tag{S21}$$

and

$$\mathrm{tr}\left[\hat{\rho}(t)\hat{\mathcal{O}}\right] = \mathrm{tr}\left[\hat{\rho}_0\hat{\mathcal{O}}\right] - \frac{i}{\hbar}\int_{-\infty}^{t} \mathrm{tr}\left[[\mathcal{V}_D(t'), \hat{\rho}_0]\hat{\mathcal{O}}_D(t)\right]\mathrm{d}t'. \tag{S22}$$

Furthermore, we can manipulate this expression and identify that

$$\mathrm{tr}\left[\hat{\rho}(t)\hat{\mathcal{O}}\right] = \mathrm{tr}\left[\hat{\rho}_0\hat{\mathcal{O}}\right] - \frac{i}{\hbar}\int_{-\infty}^{t} \mathrm{tr}\left[\hat{\rho}_0[\hat{\mathcal{O}}_D(t), \mathcal{V}_D(t')]\right]\mathrm{d}t'. \tag{S23}$$

This shows that

$$\left\langle\hat{\mathcal{O}}\right\rangle = \left\langle\hat{\mathcal{O}}\right\rangle_0 - \frac{i}{\hbar}\int_{-\infty}^{t} \left\langle[\hat{\mathcal{O}}_D(t), \mathcal{V}_D(t')]\right\rangle_0 \mathrm{d}t'. \tag{S24}$$

Finally, we can find the time-dependent induced modification by the weak perturbation

$$\Delta\hat{\mathcal{O}}(t) = \left\langle\hat{\mathcal{O}}\right\rangle - \left\langle\hat{\mathcal{O}}\right\rangle_0 = -\frac{i}{\hbar}\int_{-\infty}^{t} \left\langle[\hat{\mathcal{O}}_D(t), \mathcal{V}_D(t')]\right\rangle_0 \mathrm{d}t', \tag{S25}$$

and this is known as *the general Kubo formula* in literature [1, 2].

Now, using this general Kubo formula we can derive an expression for the current response for a weak electric field. The perturbation operator corresponding to the weak electric field is the position space integral over the current operator $\hat{\mathbf{j}}(\mathbf{r}, t)$ and the vector potential $\mathbf{A}(\mathbf{r}, t)$ of the applied probe field [2]

$$\mathcal{V}_D(t) = -\int_V \hat{\mathbf{j}}(\mathbf{r}, t)\cdot\mathbf{A}(\mathbf{r}, t)\ \mathrm{d}^3\mathbf{r}, \tag{S26}$$

where V is the 3-dimensional system volume. This contains the interaction between total electric field and the particles of the system. Selecting the Coulomb gauge where $\nabla\cdot\mathbf{A}(\mathbf{r}, t) = 0$, we can assume the scalar potential of the electric field is zero. Furthermore, for N particle system, the current operator can be written as [2]

$$\hat{\mathbf{j}}(\mathbf{r}, t) = \frac{e}{2m}\sum_{\iota=1}^{N}[\hat{\mathbf{p}}_\iota(t)\delta(\mathbf{r}-\mathbf{r}_\iota) + \delta(\mathbf{r}-\mathbf{r}_\iota)\hat{\mathbf{p}}_\iota(t)]. \tag{S27}$$

Here, $\hat{\mathbf{p}}_\iota(t)$ is the momentum operator for ι-th electron, e is the electron charge, and the m is the effective electron mass.

Induced current $\hat{\mathbf{J}}(\mathbf{r},t)$ can be introduced with stating the distinction between induced current operator $\hat{\mathbf{J}}(\mathbf{r},t)$ and the current operator $\hat{\mathbf{j}}(\mathbf{r},t)$. The $\hat{\mathbf{j}}(\mathbf{r},t)$ is used in the Hamiltonian of the quantum description, while $\hat{\mathbf{J}}(\mathbf{r},t)$ is the actual current measured by the experimentalists [2]. The expectation value of the current in the experiments can be represented as an average value for the velocity of particles that contribute to charge transport in the system, which is taken as the summation over all the particle velocities divided by the system volume:

$$\left\langle \hat{\mathbf{J}}(\mathbf{r},t)\right\rangle = \frac{e}{V}\left\langle \sum_{\iota=1}^{N} \hat{\mathbf{v}}_\iota(t)\delta(\mathbf{r}-\mathbf{r}_\iota)\right\rangle = \frac{e}{V}\sum_{\iota=1}^{N}\langle \hat{\mathbf{v}}_\iota(t)\rangle. \tag{S28}$$

Under an externally applied vector potential, the canonical velocity of a particle in a quantum system can be represented as [1]

$$\hat{\mathbf{v}}_\iota(t) = \frac{1}{m}\left[\hat{\mathbf{p}}_\iota(t) - e\mathbf{A}(\mathbf{r},t)\right], \tag{S29}$$

and substituting equation (S29) back into equation (S28), we can observe that

$$\left\langle \hat{\mathbf{J}}(\mathbf{r},t)\right\rangle = \frac{e}{mV}\sum_{\iota=1}^{N}\langle \hat{\mathbf{p}}_\iota(t)\rangle - \frac{e^2}{mV}\sum_{\iota=1}^{N}\langle \mathbf{A}(\mathbf{r},t)\rangle. \tag{S30}$$

Assuming the applied external field has a large wavelength compare with the test system's dimensions, we can consider constant vector potential over all the particles, and this leads to

$$\left\langle \hat{\mathbf{J}}(\mathbf{r},t)\right\rangle = \frac{e}{mV}\sum_{\iota=1}^{N}\langle \hat{\mathbf{p}}_\iota(t)\rangle - \frac{e^2}{mV}\sum_{\iota=1}^{N}\mathbf{A}(\mathbf{r},t). \tag{S31}$$

Introducing current operator relation with momentum operator [2]

$$\hat{\mathbf{j}}_\iota(\mathbf{r},t) = \frac{e}{m}\hat{\mathbf{p}}_\iota(\mathbf{r},t), \tag{S32}$$

and particle density in the system

$$n = \frac{N}{V}, \tag{S33}$$

we can simplify the equation (S31), and obtain

$$\left\langle \hat{\mathbf{J}}(\mathbf{r},t)\right\rangle = \frac{1}{V}\sum_{\iota=1}^{N}\left\langle \hat{\mathbf{j}}_\iota(\mathbf{r},t)\right\rangle - \frac{e^2 n}{m}\mathbf{A}(\mathbf{r},t). \tag{S34}$$

Then, assuming we can find one particle in unit volume, we can more simplify above expression into

$$\left\langle \hat{\mathbf{J}}(\mathbf{r},t)\right\rangle = \left\langle \hat{\mathbf{j}}(\mathbf{r},t)\right\rangle - \frac{e^2 n}{m}\mathbf{A}(\mathbf{r},t), \tag{S35}$$

where $\left\langle \hat{\mathbf{j}}(\mathbf{r},t)\right\rangle$ is the expected value of the current operator that can be realized using the Kubo formula. For the sake of simplicity only, this equation can be restricted to one-direction and obtain

$$\left\langle \hat{J}^a(\mathbf{r},t)\right\rangle = \left\langle \hat{j}^a(\mathbf{r},t)\right\rangle - \frac{e^2 n}{m}A^a(\mathbf{r},t), \tag{S36}$$

where $a \in \{x,y,z\}$ is the directional vector component in position space.

Next, we can apply the general Kubo formula to the current operator and derive that

$$\left\langle \hat{j}^a(\mathbf{r},t)\right\rangle = \left\langle \hat{j}^a(\mathbf{r},t_0)\right\rangle_0 - \frac{i}{\hbar}\int_{-\infty}^{t} \left\langle [\hat{j}^a(\mathbf{r},t), \mathcal{V}_D(t')]\right\rangle_0 \mathrm{d}t', \tag{S37}$$

where

$$\mathcal{V}_D(t') = -\int_V \hat{\mathbf{j}}(\mathbf{r}',t')\cdot\mathbf{A}(\mathbf{r}',t')\ \mathrm{d}^3\mathbf{r}', \tag{S38}$$

and

$$\mathcal{V}_D(t') = -\sum_b \int_V \hat{j}^b(\mathbf{r}',t')A^b(\mathbf{r}',t')\ \mathrm{d}^3\mathbf{r}'. \tag{S39}$$

Since there is no current in the solid in the absence of an electric field or some equivalent such as a time-varying magnetic field

$$\left\langle \hat{j}^a(\mathbf{r},t_0)\right\rangle_0 = 0. \tag{S40}$$

Thus, we can identify that

$$\left\langle \hat{j}^a(\mathbf{r},t)\right\rangle = \frac{i}{\hbar}\sum_b \int_{-\infty}^{t}\int_V \left\langle [\hat{j}^a(\mathbf{r},t), \hat{j}^b(\mathbf{r}',t')]\right\rangle_0 A^b(\mathbf{r}',t')\ \mathrm{d}^3\mathbf{r}'\mathrm{d}t'. \tag{S41}$$

Then, we can define the current response function as

$$\mathcal{G}^{ab}(\mathbf{r}-\mathbf{r}',t-t') = \frac{i}{\hbar}\Theta(t-t')\left\langle [\hat{j}^a(\mathbf{r},t), \hat{j}^b(\mathbf{r}',t')]\right\rangle_0, \tag{S42}$$

where $\Theta(t)$ is the Heaviside function. This leads to

$$\left\langle \hat{j}^a(\mathbf{r},t)\right\rangle = \sum_b \int_{-\infty}^{\infty}\int_V \mathcal{G}^{ab}(\mathbf{r}-\mathbf{r}',t-t')A^b(\mathbf{r}',t')\ \mathrm{d}^3\mathbf{r}'\mathrm{d}t'. \tag{S43}$$

For simplicity, we can transform this into the momentum and frequency domain as follows

$$\left\langle \hat{j}^a(\mathbf{k},\omega)\right\rangle = \frac{1}{V}\int_{-\infty}^{\infty}\int_V \left\langle \hat{j}^a(\mathbf{r},t)\right\rangle \exp(-\mathbf{k}\cdot\mathbf{r})\exp(i\omega t)\ \mathrm{d}^3\mathbf{r}\mathrm{d}t, \tag{S44}$$

and this can be evaluated as

$$\left\langle \hat{j}^a(\mathbf{k},\omega)\right\rangle = \frac{1}{V}\sum_b \int_{-\infty}^{\infty}\int_V \int_{-\infty}^{\infty}\int_V \mathcal{G}^{ab}(\mathbf{r}-\mathbf{r}',t-t')A^b(\mathbf{r}',t')\exp(-\mathbf{k}\cdot\mathbf{r})\exp(i\omega t)\ \mathrm{d}^3\mathbf{r}'\mathrm{d}t'\mathrm{d}^3\mathbf{r}\mathrm{d}t. \tag{S45}$$

Using the convolution theorem in the Fourier transform[1], we can identify that

$$\left\langle \hat{j}^a(\mathbf{k},\omega)\right\rangle = \frac{1}{V}\sum_b \int_{-\infty}^{\infty}\int_{-\infty}^{\infty} \mathcal{G}^{ab}(\mathbf{k},t-t')A^b(\mathbf{k},t')\exp(i\omega t)\ \mathrm{d}t'\mathrm{d}t. \tag{S46}$$

It is important to note that

$$\mathcal{G}^{ab}(\mathbf{k},t-t') = \left\langle [\hat{j}^a(\mathbf{k},t), \hat{j}^b(-\mathbf{k},t')]\right\rangle_0. \tag{S47}$$

Then, this leads to

$$\left\langle \hat{j}^a(\mathbf{k},\omega)\right\rangle = \frac{i}{\hbar V}\sum_b \int_{-\infty}^{\infty}\int_{-\infty}^{\infty} \Theta(t-t')\left\langle [\hat{j}^a(\mathbf{k},t), \hat{j}^b(-\mathbf{k},t')]\right\rangle_0 A^b(\mathbf{k},t')\exp(i\omega t)\ \mathrm{d}t'\mathrm{d}t. \tag{S48}$$

Finally, combining the previous derivation of experimental average current measurement in equation (S36) and the latest finding on the statistical thermal expected value of current operator, we can identify the Kubo formula for linear current response to a probe electric field as

$$\left\langle \hat{j}^a(\mathbf{k},\omega)\right\rangle = \frac{i}{\hbar V}\sum_b \int_{-\infty}^{\infty}\int_{-\infty}^{\infty} \theta(t-t')\left\langle [\hat{j}^a(\mathbf{k},t), \hat{j}^b(-\mathbf{k},t')]\right\rangle_0 A^b(\mathbf{k},t')\exp(i\omega t)\,\mathrm{d}t'\mathrm{d}t - \frac{e^2 n}{m}A^a(\mathbf{k},\omega). \tag{S49}$$

Then, adapting the integral representation of the step function

$$\theta(t) = \lim_{\mu\to 0^+}\frac{i}{2\pi}\int_{-\infty}^{\infty}\frac{\exp(-i\omega t)}{\omega + i\mu}\mathrm{d}\omega, \tag{S50}$$

the Kubo formula for the current response can be derived as

$$\left\langle \hat{j}^a(\mathbf{k},\omega)\right\rangle = \lim_{\mu\to 0^+} -\frac{1}{2\pi\hbar V}\sum_b \int_{-\infty}^{\infty}\int_{-\infty}^{\infty}\int_{-\infty}^{\infty} \mathcal{G}^{ab}(\mathbf{k},t-t')A^b(\mathbf{k},t')\exp(i\omega t)\frac{\exp(-i\omega'(t-t'))}{\omega'+i\mu}\mathrm{d}\omega'\mathrm{d}t'\mathrm{d}t - \frac{e^2 n}{m}A^a(\mathbf{k},\omega). \tag{S51}$$

1.2 The Floquet picture of the Kubo formula

Now, we consider a metallic system subjected to a high-intensity dressing field. The wave function of a single electron in the dressed metal can be identified as Floquet states [3]. Due to the presence of high-intensity external dressing in our case, the system will not be in equilibrium. However, in what follows one can assume the system to be in a stationary state so that the occupation number of Floquet states are independent [4].

From second quantization formalism, the current operators $\hat{j}^{a,b}(\mathbf{k},t)$ can expand using the Floquet states [3] as the basis for our system as follows

$$\hat{j}^a(\mathbf{k},t) = \sum_{\alpha\beta}\hat{j}^a_{\alpha\beta}(\mathbf{k},t)\hat{a}^\dagger_\alpha(t_0=0)\hat{a}_\beta(t_0=0), \tag{S51}$$

where $\hat{a}^\dagger_\alpha(t)$ and $\hat{a}_\alpha(t)$ are creation and annihilation operators for α-th Floquet state $\left|\psi_\alpha\right\rangle$. These operators should satisfy the following relationships

$$\hat{a}^\dagger_\alpha(t)\left|0\right\rangle = \left|\psi_\alpha(t)\right\rangle, \quad \hat{a}_\alpha(t)\left|0\right\rangle = 0, \tag{S52}$$

$$[\hat{a}_\alpha(t), \hat{a}^\dagger_\beta(t)]_\pm = \delta_{\alpha\beta}, \quad [\hat{a}_\alpha(t), \hat{a}_\beta(t)]_\pm = [\hat{a}^\dagger_\alpha(t), \hat{a}^\dagger_\beta(t)]_\pm = 0. \tag{S53}$$

Here, $\left|0\right\rangle$ is the vacuum state containing no particle, and positive (negative) subscripts refer to the fermionic anticommutators (commutators) for the Floquet state particles. Furthermore, $\hat{j}^a_{\alpha\beta}(\mathbf{k},t)$ is the single particle matrix element given in the Schrödinger's picture

$$\hat{j}^a_{\alpha\beta}(\mathbf{k},t) = \left\langle \psi_\alpha(t)\middle|\hat{j}^a(\mathbf{k})\middle|\psi_\beta(t)\right\rangle \tag{S54}$$

where $\hat{j}^a(\mathbf{k}) = \hat{j}^a(\mathbf{k},t=0)$. Using the properties of the Floquet states, we can derive that

$$\hat{j}^a_{\alpha\beta}(\mathbf{k},t) = \left\langle u_\alpha(t)\middle|\exp\left(\frac{i\epsilon_\alpha t}{\hbar}\right)\hat{j}^a(\mathbf{k})\exp\left(-\frac{i\epsilon_\beta t}{\hbar}\right)\middle|u_\beta(t)\right\rangle. \tag{S55}$$

Here, ϵ_α is the quasienergy, and $|u_\alpha(t)\rangle$ is the time-periodic Floquet mode of the α-th Floquet state. Considering the Fourier series expand of the time-periodic Floquet modes, we can identify that

$$\hat{j}^a_{\alpha\beta}(\mathbf{k},t) = \sum_{n_1,n_2=-\infty}^{\infty} \exp\left\{\frac{i}{\hbar}[(\epsilon_\alpha - \epsilon_\beta) + (n_1 - n_2)\hbar\Omega]t\right\}\left\langle u_\alpha^{n_1}\middle|\hat{j}^a(\mathbf{k})\middle|u_\beta^{n_2}\right\rangle, \tag{S56}$$

where Ω is the external dressing field's angular frequency that generates the Floquet states, and n_1, n_2 are integers.

Next, we can evaluate the commutator operator in equation (S47) using the above derived second quantization expansion as follows

$$[\hat{j}^a(\mathbf{k},t), \hat{j}^b(-\mathbf{k},t')] = \sum_{\alpha\beta\eta\rho} \hat{j}^a_{\alpha\beta}(\mathbf{k},t)\hat{j}^a_{\eta\rho}(-\mathbf{k},t')[\hat{a}^\dagger_\alpha \hat{a}_\beta, a^\dagger_\eta a_\rho], \tag{S57}$$

and this can be simplified to

$$[\hat{j}^a(\mathbf{k},t), \hat{j}^b(-\mathbf{k},t')] = \sum_{\alpha\beta} \hat{j}^a_{\alpha\beta}(\mathbf{k},t)\hat{j}^a_{\beta\alpha}(-\mathbf{k},t')(\hat{a}^\dagger_\alpha \hat{a}_\alpha - \hat{a}^\dagger_\beta \hat{a}_\beta). \tag{S58}$$

The statistical thermal average of this commutator is evaluated concerning the aforementioned time-independent non-equilibrium density matrix as mentioned in equation (S47). Here, $\hat{a}^\dagger_\alpha \hat{a}_\alpha$ represent the number operator for the α-th Floquet state. Thus, we can introduce the distribution functions for the Floquet state particles in the system as follows

$$\mathcal{F}_\alpha := \left\langle \hat{a}^\dagger_\alpha \hat{a}_\alpha \right\rangle, \quad \mathcal{F}_\beta := \left\langle \hat{a}^\dagger_\beta \hat{a}_\beta \right\rangle. \tag{S59}$$

It is important to note that these distribution functions not necessarily to be equilibrium distribution functions, however it is assumed that these are time-independent. Thus, the statistical thermal expectation value of the above commutator becomes

$$\begin{aligned}\mathcal{G}^{ab}(\mathbf{k},t-t') &= \left\langle [\hat{j}^a(\mathbf{k},t), \hat{j}^b(-\mathbf{k},t')] \right\rangle_0 \\ &= \sum_{\alpha\beta} \sum_{n_1,\dots,n_4=-\infty}^{\infty} \exp\left\{\frac{i}{\hbar}[(\epsilon_\alpha - \epsilon_\beta) + (n_1 - n_2)\hbar\Omega]t\right\} \exp\left\{\frac{i}{\hbar}[(\epsilon_\beta - \epsilon_\alpha) + (n_3 - n_4)\hbar\Omega]t\right\} \\ &\quad \times \left\langle u_\alpha^{n_1}\middle|\hat{j}^a(\mathbf{k})\middle|u_\beta^{n_2}\right\rangle \left\langle u_\beta^{n_3}\middle|\hat{j}^b(-\mathbf{k})\middle|u_\alpha^{n_4}\right\rangle (\mathcal{F}_\alpha - \mathcal{F}_\beta).\end{aligned} \tag{S60}$$

Remark that the operator $\hat{j}^a(\mathbf{k})$ is the single-particle current operator in momentum domain and that can be defined as

$$\hat{j}^a(\mathbf{k}) = \frac{-e}{2m}[\hat{\mathbf{p}}^a \exp(-i\mathbf{k}\cdot\mathbf{r}) + \exp(-i\mathbf{k}\cdot\mathbf{r})\hat{\mathbf{p}}^a]. \tag{S61}$$

Finally, combining these derivations back into the Kubo formula presented in equation (S51), we can identify that

$$\begin{aligned}\left\langle \hat{j}^a(\mathbf{k},\omega) \right\rangle &= \lim_{\mu\to 0^+} -\frac{1}{2\pi\hbar V} \sum_b \int_{-\infty}^{\infty}\int_{-\infty}^{\infty}\int_{-\infty}^{\infty} \sum_{\alpha\beta} \sum_{n_1,\dots,n_4=-\infty}^{\infty} \\ &\quad \times \exp\left\{i\left[\omega - \omega' + \frac{1}{\hbar}(\epsilon_\alpha - \epsilon_\beta) + (n_1 - n_2)\Omega\right]t\right\} \exp\left\{i\left[\omega' - \frac{1}{\hbar}(\epsilon_\alpha - \epsilon_\beta) + (n_3 - n_4)\Omega\right]t'\right\} \\ &\quad \times \left\langle u_\alpha^{n_1}\middle|\hat{j}^a(\mathbf{k})\middle|u_\beta^{n_2}\right\rangle \left\langle u_\beta^{n_3}\middle|\hat{j}^b(-\mathbf{k})\middle|u_\alpha^{n_4}\right\rangle A^b(\mathbf{k},t') \frac{\left(\mathcal{F}_\alpha - \mathcal{F}_\beta\right)}{\omega' + i\mu}\, \mathrm{d}\omega' \mathrm{d}t' \mathrm{d}t \\ &\quad - \frac{e^2 n}{m} A^a(\mathbf{k},\omega).\end{aligned} \tag{S62}$$

Then, we can expand the vector potential corresponding to the probe bias in the frequency domain as follows

$$A^b(\mathbf{k},t') = \frac{1}{2\pi}\int_{-\infty}^{\infty} A^b(\mathbf{k},\omega'')\exp(-i\omega''t')\,\mathrm{d}\omega'', \tag{S63}$$

and submitting this back into the Kubo formula we get

$$\begin{aligned}
\left\langle \hat{J}^a(\mathbf{k},\omega)\right\rangle = &\lim_{\mu\to 0^+} -\frac{1}{4\pi^2\hbar V}\sum_b \int_{-\infty}^{\infty}\int_{-\infty}^{\infty}\int_{-\infty}^{\infty}\int_{-\infty}^{\infty}\sum_{\alpha\beta}\sum_{n_1,\dots,n_4=-\infty}^{\infty} \\
&\times \exp\left\{ i\left[\omega-\omega'+\frac{1}{\hbar}(\epsilon_\alpha-\epsilon_\beta)+(n_1-n_2)\Omega\right]t\right\}\exp\left\{ i\left[\omega'-\omega''-\frac{1}{\hbar}(\epsilon_\alpha-\epsilon_\beta)+(n_3-n_4)\Omega\right]t'\right\} \\
&\times \left\langle u_\alpha^{n_1}\middle|\hat{j}^a(\mathbf{k})\middle|u_\beta^{n_2}\right\rangle\left\langle u_\beta^{n_3}\middle|\hat{j}^b(-\mathbf{k})\middle|u_\alpha^{n_4}\right\rangle A^b(\mathbf{k},\omega'')\frac{\left(\mathcal{F}_\alpha-\mathcal{F}_\beta\right)}{\omega'+i\mu}\,\mathrm{d}\omega'\mathrm{d}\omega''\mathrm{d}t'\mathrm{d}t \\
&-\frac{e^2 n}{m}A^a(\mathbf{k},\omega).
\end{aligned} \tag{S64}$$

By evaluating the time integrals and ω' integral, we can simply this formula into

$$\begin{aligned}
\left\langle \hat{J}^a(\mathbf{k},\omega)\right\rangle = &\lim_{\mu\to 0^+} -\frac{1}{\hbar V}\sum_b \int_{-\infty}^{\infty}\sum_{\alpha\beta}\sum_{n_1,\dots,n_4=-\infty}^{\infty} \\
&\times \frac{\delta\left(\omega-\omega''+(n_1-n_2+n_3-n_4)\Omega\right)}{\left[\omega+\frac{1}{\hbar}(\epsilon_\alpha-\epsilon_\beta)+(n_1-n_2)\Omega+i\mu\right]} \\
&\times \left\langle u_\alpha^{n_1}\middle|\hat{j}^a(\mathbf{k})\middle|u_\beta^{n_2}\right\rangle\left\langle u_\beta^{n_3}\middle|\hat{j}^b(-\mathbf{k})\middle|u_\alpha^{n_4}\right\rangle A^b(\mathbf{k},\omega'')\left(\mathcal{F}_\alpha-\mathcal{F}_\beta\right)\mathrm{d}\omega'' \\
&-\frac{e^2 n}{m}A^a(\mathbf{k},\omega).
\end{aligned} \tag{S65}$$

We can specify the probe electric field relation with vector potential using the electromagnetic theory

$$A^b(\mathbf{k},\omega) = -\frac{i}{(\omega+i\gamma)}E^b(\mathbf{k},\omega). \tag{S66}$$

Here, the factor γ is a phenomenological way to include scattering damping effects in the quantum calculations. Then, we can re-write the Kubo formulas as follows

$$\begin{aligned}
\left\langle \hat{J}^a(\mathbf{k},\omega)\right\rangle = &\lim_{\mu\to 0^+} \frac{i}{\hbar V}\sum_b \int_{-\infty}^{\infty}\sum_{\alpha\beta}\sum_{n_1,\dots,n_4=-\infty}^{\infty} \\
&\times \frac{\delta\left(\omega-\omega''+(n_1-n_2+n_3-n_4)\Omega\right)}{\left[\omega+\frac{1}{\hbar}(\epsilon_\alpha-\epsilon_\beta)+(n_1-n_2)\Omega+i\mu\right]} \\
&\times \left\langle u_\alpha^{n_1}\middle|\hat{j}^a(\mathbf{k})\middle|u_\beta^{n_2}\right\rangle\left\langle u_\beta^{n_3}\middle|\hat{j}^b(-\mathbf{k})\middle|u_\alpha^{n_4}\right\rangle E^b(\mathbf{k},\omega'')\frac{\left(\mathcal{F}_\alpha-\mathcal{F}_\beta\right)}{\omega+i\gamma}\,\mathrm{d}\omega'' \\
&+\frac{ie^2 n}{m(\omega+i\gamma)}E^b(\mathbf{k},\omega''),
\end{aligned} \tag{S67}$$

and this can restructure as

$$\left\langle \hat{j}^a(\mathbf{k},\omega)\right\rangle = \lim_{\mu\to 0^+}\sum_b\int_{-\infty}^{\infty}\left\{\frac{i}{\hbar V}\sum_{\alpha\beta}\sum_{n_1,\ldots,n_4=-\infty}^{\infty}\frac{\delta\left(\omega-\omega''+(n_1-n_2+n_3-n_4)\Omega\right)}{\left[\omega+\frac{1}{\hbar}(\epsilon_\alpha-\epsilon_\beta)+(n_1-n_2)\Omega+i\mu\right]}\right.$$
$$\left.\times\left\langle u_\alpha^{n_1}\middle|\hat{j}^a(\mathbf{k})\middle|u_\beta^{n_2}\right\rangle\left\langle u_\beta^{n_3}\middle|\hat{j}^b(-\mathbf{k})\middle|u_\alpha^{n_4}\right\rangle\frac{\left(\mathcal{F}_\alpha-\mathcal{F}_\beta\right)}{\omega+i\gamma}+\frac{ie^2n}{m(\omega+i\gamma)}\delta(\omega-\omega'')\delta_{ab}\right\}E^b(\mathbf{k},\omega'')\,\mathrm{d}\omega''. \tag{S68}$$

By initiating the conductivity tensor

$$\sigma^{ab}(\mathbf{k},\omega,\omega'') := \lim_{\mu\to 0^+}\frac{i}{\hbar V}\sum_{\alpha\beta}\sum_{n_1,\ldots,n_4=-\infty}^{\infty}\frac{\delta\left(\omega-\omega''+(n_1-n_2+n_3-n_4)\Omega\right)}{\left[\omega+\frac{1}{\hbar}(\epsilon_\alpha-\epsilon_\beta)+(n_1-n_2)\Omega+i\mu\right]}$$
$$\times\left\langle u_\alpha^{n_1}\middle|\hat{j}^a(\mathbf{k})\middle|u_\beta^{n_2}\right\rangle\left\langle u_\beta^{n_3}\middle|\hat{j}^b(-\mathbf{k})\middle|u_\alpha^{n_4}\right\rangle\frac{\left(\mathcal{F}_\alpha-\mathcal{F}_\beta\right)}{\omega+i\gamma}+\frac{ie^2n}{m(\omega+i\gamma)}\delta(\omega-\omega'')\delta_{ab}, \tag{S68}$$

and we can rewrite the Kubo formula in a compact form

$$\left\langle \hat{j}^a(\mathbf{k},\omega)\right\rangle = \sum_b\int_{-\infty}^{\infty}\sigma^{ab}(\mathbf{k},\omega,\omega'')E^b(\mathbf{k},\omega'')\,\mathrm{d}\omega''. \tag{S69}$$

Above derived expression implies that the current is, as opposed to the un-driven case, no longer a simple product of conductivity and perturbation electric field, since it is convoluted over the bias frequency ω''. It is significant to note that the conductivity tensor introduced previously, depends on both the response frequency ω and the bias frequency ω''.

Following the general derivation, we can restrict the conditions which are acting on the parameters concerning our Floquet system. The response and bias frequency ω and ω'' are assumed to be in the central Floquet zone

$$|\omega|,|\omega''|<|\frac{\Omega}{2}| \quad\Rightarrow\quad |\omega-\omega''|<\Omega. \tag{S70}$$

Under this condition, we can identify that the delta distribution in equation (S68) only can be non-zero with the condition

$$n_1-n_2+n_3-n_4=0. \tag{S71}$$

Thus, this leads to ω''-independent conductivity tensor

$$\sigma^{ab}(\mathbf{k},\omega) = \lim_{\mu\to 0^+}\frac{i}{\hbar V(\omega+i\gamma)}\sum_{\alpha\beta}\sum_{n_1,\ldots,n_4=-\infty}^{\infty}\frac{\left(\mathcal{F}_\alpha-\mathcal{F}_\beta\right)}{\left[\omega+\frac{1}{\hbar}(\epsilon_\alpha-\epsilon_\beta)+(n_1-n_2)\Omega+i\mu\right]}$$
$$\times\left\langle u_\alpha^{n_1}\middle|\hat{j}^a(\mathbf{k})\middle|u_\beta^{n_2}\right\rangle\left\langle u_\beta^{n_3}\middle|\hat{j}^b(-\mathbf{k})\middle|u_\alpha^{n_4}\right\rangle+\frac{ie^2n}{m(\omega+i\gamma)}\delta_{ab}. \tag{S72}$$

Concentrate on more special case of conductivity by analyzing only the longitudinal conductivity where $a=b=x$, and we can obtain that

$$\sigma^{xx}(\mathbf{k},\omega) = \lim_{\mu\to 0^+}\frac{i}{\hbar V(\omega+i\gamma)}\sum_{\alpha\beta}\sum_{n_1,\ldots,n_4=-\infty}^{\infty}\frac{\left(\mathcal{F}_\alpha-\mathcal{F}_\beta\right)}{\left[\omega+\frac{1}{\hbar}(\epsilon_\alpha-\epsilon_\beta)+(n_1-n_2)\Omega+i\mu\right]}$$
$$\times\left\langle u_\alpha^{n_1}\middle|\hat{j}^x(\mathbf{k})\middle|u_\beta^{n_2}\right\rangle\left\langle u_\beta^{n_3}\middle|\hat{j}^x(-\mathbf{k})\middle|u_\alpha^{n_4}\right\rangle+\frac{ie^2n}{m(\omega+i\gamma)}. \tag{S72}$$

By assuming that the current response is spatially homogeneous, we can assume that $\mathbf{k} \to 0$, and we can obtain

$$\sigma^{xx}(0,\omega) = \lim_{\mu\to 0^+} \frac{i}{\hbar V(\omega + i\gamma)} \sum_{\alpha\beta} \sum_{n_1,\ldots,n_4=-\infty}^{\infty} \frac{\left(\mathcal{F}_\alpha - \mathcal{F}_\beta\right)}{\left[\omega + \frac{1}{\hbar}(\epsilon_\alpha - \epsilon_\beta) + (n_1 - n_2)\Omega + i\mu\right]} \times \left\langle u_\alpha^{n_1} \middle| \hat{j}^x(0) \middle| u_\beta^{n_2} \right\rangle \left\langle u_\beta^{n_3} \middle| \hat{j}^x(0) \middle| u_\alpha^{n_4} \right\rangle + \frac{ie^2 n}{m(\omega + i\gamma)}, \tag{S73}$$

where

$$\hat{j}^x(0) = \frac{-e}{2m}(\hat{p}^x \exp(0) + \exp(0)\hat{p}^x) = \frac{-e}{m}\hat{p}^x. \tag{S74}$$

Now, we can derive a simplified version of the conductivity tensor

$$\sigma^{xx}(\omega) = \lim_{\mu\to 0^+} \frac{i}{\hbar V(\omega + i\gamma)} \frac{e^2}{m^2} \sum_{\alpha\beta} \sum_{n_1,\ldots,n_4=-\infty}^{\infty} \frac{\left(\mathcal{F}_\alpha - \mathcal{F}_\beta\right)}{\left[\omega + \frac{1}{\hbar}(\epsilon_\alpha - \epsilon_\beta) + (n_1 - n_2)\Omega + i\mu\right]} \times \left\langle u_\alpha^{n_1} \middle| \hat{p}^x \middle| u_\beta^{n_2} \right\rangle \left\langle u_\beta^{n_3} \middle| \hat{p}^x \middle| u_\alpha^{n_4} \right\rangle + \frac{ie^2 n}{m(\omega + i\gamma)}, \tag{S75}$$

The conductivity tensor describes the physically indistinguishable effects of polarization and current response. Thus, it is possible in general to express susceptibility as a function of linear conductivity [5]

$$\chi^{xx}(\omega) = \frac{\sigma^{xx}(\omega)}{-i\omega}. \tag{S76}$$

Then, we can identify the susceptibility function of the dressed quantum system as

$$\chi^{xx}(\omega) = \lim_{\mu\to 0^+} \frac{1}{\hbar\omega(\omega + i\gamma)} \frac{e^2}{m^2 V} \sum_{\alpha\beta} \sum_{n_1,\ldots,n_4=-\infty}^{\infty} \frac{\left(\mathcal{F}_\alpha - \mathcal{F}_\beta\right)}{\left[\omega + \frac{1}{\hbar}(\epsilon_\alpha - \epsilon_\beta) + (n_1 - n_2)\Omega + i\mu\right]} \times \left\langle u_\alpha^{n_1} \middle| \hat{p}^x \middle| u_\beta^{n_2} \right\rangle \left\langle u_\beta^{n_3} \middle| \hat{p}^x \middle| u_\alpha^{n_4} \right\rangle + \frac{e^2 n}{m\omega(\omega + i\gamma)}. \tag{S77}$$

1.3 Susceptibility function of dressed metallic system

In the previous subsection, we derived a general expression for the susceptibility function of dressed quantum system. However, under our study we consider a dressed metallic system. Here, we used the free electron model to describe the transport properties of the dressed metallic system. Thus, we can use the Fermi-Dirac distribution as the particle distribution functions, and we can express the Fermi-Dirac distribution as

$$\mathcal{F}(\epsilon) = \frac{1}{\exp\left(\frac{\epsilon - \epsilon_F}{k_B T}\right) + 1}. \tag{S77}$$

Furthermore, we consider very low-temperature conditions ($T \to 0$), and we can re-write the Fermi-Dirac distribution as

$$\mathcal{F}(\epsilon) = \lim_{T\to 0} \frac{1}{\exp\left(\frac{\epsilon - \epsilon_F}{k_B T}\right) + 1} \approx \Theta(\epsilon_F - \epsilon), \tag{S78}$$

where, ϵ_F is the Fermi energy, k_B is the Boltzmann constant, and T is the absolute temperature. Now, we can restructure the general susceptibility function according to the dressed metallic system

$$\begin{aligned}\chi^{xx}(\omega) = \lim_{\mu\to 0^+} \frac{1}{\hbar\omega(\omega+i\gamma)} \frac{e^2}{m^2 V} \sum_{\alpha\beta} \sum_{n_1,\ldots,n_4=-\infty}^{\infty} \frac{\left(\Theta(\epsilon_F-\epsilon_\alpha)-\Theta(\epsilon_F-\epsilon_\beta)\right)}{\left[\omega+\frac{1}{\hbar}(\epsilon_\alpha-\epsilon_\beta)+(n_1-n_2)\Omega+i\mu\right]} \\ \times \left\langle u_\alpha^{n_1}\middle|\hat{p}^x\middle|u_\beta^{n_2}\right\rangle \left\langle u_\beta^{n_3}\middle|\hat{p}^x\middle|u_\alpha^{n_4}\right\rangle + \frac{e^2 n}{m\omega(\omega+i\gamma)}.\end{aligned} \tag{S79}$$

Since we consider the transport properties of the metallic electrons, we only need to analyze the behavior of the conduction electrons. Thus, we can assume that we have to consider the electron that have energy of Fermi energy. This leads to assume that $\epsilon_\alpha = \epsilon'$, and $\epsilon_\beta = \epsilon''$, where $\epsilon', \epsilon'' \to \epsilon_F$. Next, we can apply these changes back into the susceptibility function and derive

$$\begin{aligned}\chi^{xx}(\omega) = \lim_{\mu\to 0^+} \frac{1}{\hbar\omega(\omega+i\gamma)} \frac{e^2}{m^2 V} \lim_{\epsilon',\epsilon''\to\epsilon_F} \sum_{\alpha\beta} \sum_{n_1,\ldots,n_4=-\infty}^{\infty} \frac{(\Theta(\epsilon_F-\epsilon')-\Theta(\epsilon_F-\epsilon''))}{\left[\omega+\frac{1}{\hbar}(\epsilon'-\epsilon'')+(n_1-n_2)\Omega+i\mu\right]} \\ \times \left\langle u_\alpha^{n_1}\middle|\hat{p}^x\middle|u_\beta^{n_2}\right\rangle \left\langle u_\beta^{n_3}\middle|\hat{p}^x\middle|u_\alpha^{n_4}\right\rangle + \frac{e^2 n}{m\omega(\omega+i\gamma)}.\end{aligned} \tag{S80}$$

In our case, we assume that $\omega \neq 0$ and $\omega < \Omega/2$. Thus, the denominator of the above expression can not assume a very small value. Therefore, we can see that the first term of the above expression goes to zero. Finally, now we can assume that there is no contribution from the first term in the susceptibility function. Moreover, we can derive final expression for the susceptibility function for a dressed metallic quantum system as

$$\chi^{xx}(\omega) \approx \frac{e^2 n}{m\omega(\omega+i\gamma)}. \tag{S81}$$

By defining the metal plasma frequency for electrons as

$$\omega_{pm} = \sqrt{\frac{e^2 n}{m}}, \tag{S82}$$

we can re-write the susceptibility function in the simplest form

$$\chi^{xx}(\omega) = \frac{\omega_{pm}^2}{\omega(\omega+i\gamma)}. \tag{S83}$$

Thus, we can identify the metal dielectric function as

$$\varepsilon^{xx}(\omega) = 1 - \chi^{xx}(\omega) = 1 - \frac{\omega_{pm}^2}{\omega(\omega+i\gamma)}. \tag{S84}$$

Although the derived expression is the same as the general Drude-Sommerfeld model description, we need to consider the effects on the damping factor γ induced by the dressing field.

2 Damping factor of dressed metals

In this section, we present an expression to evaluate the dressing field effects on the metal's damping factor. First we discuss the disorder model we used to explain the effects of the metal impurities. Next, we present the Floquet-Fermi golden rule for a dressed metallic system under the explained disorder model. Here, we follow the same steps mentioned in Refs. [6, 7] with the help of the $t-t'$ formalism. Finally, we derive an expression to describe the relationship between the metal's damping factor and the dressing field.

2.1 Impurity generated scattering potential

In this study, we model the effects of metal impurities by a scattering potential $V(\mathbf{r})$. We model this potential as a collection of N_{imp} identical impurities in a volume $\mathcal{V}$, localized at randomly distributed points $\mathbf{r}_i$. We characterize this random localized single potential as $v(\mathbf{r})$, and we can identify that

$$V(\mathbf{r}) = \sum_{i=1}^{N_{\text{imp}}} v(\mathbf{r}-\mathbf{r}_i). \tag{S85}$$

Moreover, we assume the limit $\mathcal{V} \to \infty$, while keeping the impurity density $\eta_{\text{imp}} = N_{\text{imp}}\big/\mathcal{V}$ constant. The average distance between impurities is $\eta_{\text{imp}}^{-1/d}$ where d is the space dimensionality. Furthermore, we assume that the $v(\mathbf{r})$ is a central potential, with a characteristic range r_0.

With the limit of a high density ($\eta_{\text{imp}} \to \infty$) of weakly scattering impurities ($v(\mathbf{r}) \to 0$), our impurity potential behave as the Gaussian model [8]. In addition, we can consider these single central impurity as a δ-scatter

$$v(\mathbf{r}) = v_0\delta(\mathbf{r}). \tag{S86}$$

Here, v_0 is a constant, and this leads to

$$V(\mathbf{r}) = \sum_{i=1}^{N_{\text{imp}}} v_0\delta(\mathbf{r}-\mathbf{r}_i). \tag{S87}$$

If the disorders are evenly distributed over a large system, then we can assume that the properties of the system can be described as an average over its ensemble of microscopically identical subsystems [9]. This is known as the self-averaging. To make this a good approximation, the ratio between the impurity density η_{imp}, and the electron density in the metal η_{el} should be much smaller than unity $\eta_{\text{imp}}\big/\eta_{\text{el}} \ll 1$. Therefore, it is crucial to maintain a high electron density relative to the impurities in the metal being studied. Under this condition, we can evaluate the correlation function considering only a single impurity as follows [8]

$$\langle V(\mathbf{r})V(\mathbf{r}')\rangle_{\text{imp}} = \eta_{\text{imp}} \int v(\mathbf{r}''-\mathbf{r})v(\mathbf{r}''-\mathbf{r}')\mathrm{d}\mathbf{r}''. \tag{S88}$$

Furthermore, we can identify that

$$\langle V(\mathbf{r})V(\mathbf{r}')\rangle_{\text{imp}} = \eta_{\text{imp}}v_0^2\delta(\mathbf{r}-\mathbf{r}'). \tag{S89}$$

This describes that the collisions are isotropic [8]. A random scattering impurity potential along this characteristic is called as a white noise impurity.

2.2 Floquet-Fermi golden rule

Considering the above described impurity based scattering potential model, we derive the Floquet-Fermi golden rule for a dressed metallic system with the use of the t-t' formalism. As the first step, we present the Floquet states using the t-t' formalism [10] as follows

$$\left|\psi_\alpha(t,t')\right\rangle = \exp\left(-i\frac{\epsilon_\alpha}{\hbar}t\right)\left|u_\alpha(t')\right\rangle, \tag{S90}$$

by splitting the aperiodic and periodic terms of the Floquet states. Here, α is the quantum number of the considering Floquet state. Moreover, we can recognize repeated Floquet states in each Floquet zone ($l = 0, \pm1, \pm2, \ldots$) [6, 10]. Thus, we can identify general t-t'-state in the l-th Floquet zone as

$$\left|\psi_\alpha^l(t,t')\right\rangle = \exp[il\omega(t'-t)]\left|\psi_\alpha(t,t')\right\rangle. \tag{S91}$$

Examining the characteristics of t-t'-Floquet states, we can identify that these states fulfill the t-t'-Schrödinger equation [6, 10]

$$i\hbar\frac{\partial}{\partial t}\left|\psi_\alpha^l(t,t')\right\rangle = \hat{H}_F(t')\left|\psi_\alpha^l(t,t')\right\rangle, \tag{S92}$$

where the Floquet Hamiltonian can be recognized as

$$\hat{H}_F(t') = \hat{H}_e(t') - i\hbar\frac{\partial}{\partial t'}. \tag{S93}$$

Here, $\hat{H}_e(t)$ the time-dependent Hamiltonian of the dressed many-body system [7]. Then, we can introduce the corresponding time evolution operator to the t-t'-Schrödinger equation by

$$U_0(t,t_0;t') = \exp\left(-\frac{i}{\hbar}\hat{H}_F(t')[t-t_0]\right). \tag{S94}$$

It is crucial to note that the advantage of using the t-t' formalism lies on this time evolution operator that avoids any time ordering operators [6].

Next, we model the metal impurity generated scattering potential as a group of randomly distributed impurities under the Gaussian white noise approximation. In addition, we assume that the impurity generated scattering potential $V(\mathbf{r})$ is turned on at the reference time $t = t_0$. Due to the introduced scattering potential, we should change our time-dependent Hamiltonian by adding a time-independent total perturbation

$$i\hbar\frac{\partial}{\partial t}\left|\Psi_\alpha^l(t,t')\right\rangle = \left[\hat{H}_F(t') + V(t,t';\mathbf{r})\right]\left|\Psi_\alpha^l(t,t')\right\rangle, \tag{S95}$$

where we use a new wave function solution $\left|\Psi_\alpha^l\right\rangle$ for the perturbed dressed quantum system. When $t \le t_0$, both of the solutions for equation (S92) and (S95) coincide

$$\left|\psi_\alpha^l(t,t')\right\rangle = \left|\Psi_\alpha^l(t,t')\right\rangle \quad \text{when} \quad t \le t_0. \tag{S96}$$

Applying the Dirac's interaction picture representation [1, 2], we can re-write the t-t'-Floquet state as

$$\left|\Psi_\alpha^l(t,t')\right\rangle_I = U_0^\dagger(t,t_0;t')\left|\Psi_\alpha^l(t,t')\right\rangle, \tag{S97}$$

and

$$V_I(t,t';\mathbf{r}) = U_0^\dagger(t,t_0;t')V(t,t';\mathbf{r})U_0(t,t_0;t') \tag{S98}$$

Next, we can identify the t-t'-Schrödinger equation in the interaction representation as

$$i\hbar\frac{\partial}{\partial t}\left|\Psi_\alpha^l(t,t')\right\rangle_I = V_I(t,t';\mathbf{r})\left|\Psi_\alpha^l(t,t')\right\rangle_I, \tag{S99}$$

and this equation holds recursive solutions [1, 2]

$$\left|\Psi_\alpha^l(t,t')\right\rangle_I = \left|\Psi_\alpha^l(t_0,t')\right\rangle_I + \frac{1}{i\hbar}\int_{t_0}^{t} V_I(t_1,t';\mathbf{r})\left|\Psi_\alpha^l(t_1,t')\right\rangle_I dt_1. \tag{S100}$$

We allow to assume that the impact of the high-order terms of the perturbation potential can be ignored. With the help of the Born approximation, we can approximate the solution up to the first-order term as follows

$$\left|\Psi_\alpha^l(t,t')\right\rangle_I \approx \left|\psi_\alpha^l(t_0,t')\right\rangle + \frac{1}{i\hbar}\int_{t_0}^{t} V_I(t_1,t';\mathbf{r})\left|\psi_\alpha^l(t_0,t')\right\rangle dt_1. \tag{S101}$$

We can identify that the t-t'-Floquet states always create a basis set. Thus, we can re-structure the wave function solutions for the t-t'-Schrödinger equation given in equation (S99) with the help of the previously introduced t-t'-Floquet states

$$\left|\Psi_\alpha^l(t,t')\right\rangle = \sum_\beta a_{\alpha\beta}^{ll'}(t,t')\left|\psi_\beta^{l'}(t,t')\right\rangle. \tag{S102}$$

Here, we can evaluate the coefficient by

$$\alpha_{\alpha\beta}^{ll'}(t,t') = \left\langle\psi_\beta^{l'}(t,t')\middle|\Psi_\alpha^l(t,t')\right\rangle, \tag{S103}$$

and it is known as the scattering amplitude. This leads to

$$\alpha_{\alpha\beta}^{ll'}(t,t') = \left\langle\psi_\beta^{l'}(t,t')\middle|\psi_\alpha^l(t,t')\right\rangle + \frac{1}{i\hbar}\int_{t_0}^{t}\left\langle\psi_\beta^{l'}(t_1,t')\right| V(t,t';\mathbf{r})\left|\psi_\alpha^l(t_1,t')\right\rangle dt_1. \tag{S104}$$

Next, we examine a general scattering phenomenon when an electron scatters from a previously defined t-t'-Floquet state $\left|\psi_\beta^{l'}(t,t')\right\rangle$ into a distinct perturbed t-t'-Floquet state $\left|\Psi_\alpha^l(t,t')\right\rangle$ that possesses a constant quansienergy ϵ as follows

$$\psi_\beta^{l'}(\mathbf{k}',t,t') = \mathrm{e}^{-\frac{i}{\hbar}(\epsilon_\beta(\mathbf{k}')+l'\hbar\omega)t}u_\beta(\mathbf{k}',t')\mathrm{e}^{il'\omega t'} \xrightarrow{\text{scattering}} \Psi_\alpha^l(\mathbf{k},t,t') = \mathrm{e}^{-\frac{i}{\hbar}(\epsilon+l\hbar\omega)t}\tilde{u}_\alpha(\mathbf{k},t')\mathrm{e}^{il\omega t'}. \tag{S105}$$

With the help of the expression presented in equation (S104), and assuming the scattering perturbation is a stationary potential, we can calculate the scattering amplitude for this scattering scenario

$$\alpha_{\alpha\beta}^{ll'}(t,t') = \delta_{\alpha\beta}\mathrm{e}^{i\omega(l-l')(t'-t)} + \frac{1}{i\hbar}\int_{t_0}^{t}\left\langle\psi_\beta^{l'}(t_1,t')\right| V(\mathbf{r})\left|\psi_\alpha^l(t_1,t')\right\rangle dt_1. \tag{S106}$$

As the next step, we re-write the derived scattering amplitude as a Fourier series for the second time argument t' as follows

$$\alpha_{\alpha\beta}^{ll'}(t,t') = \sum_{n=-\infty}^{\infty}\alpha_{\alpha\beta}^{ll'}(t,n)\mathrm{e}^{in\omega t'}. \tag{S107}$$

Here, the these Fourier coefficients can be represented by

$$\alpha_{\alpha\beta}^{ll'}(t,n) = \frac{1}{T}\int_0^T a_{\alpha\beta}^{ll'}(t,t')\mathrm{e}^{-in\omega t'}\mathrm{d}t'. \tag{S108}$$

Then substitute the expression from equation (S106) into equation (S108), and this leads to

$$\alpha_{\alpha\beta}^{ll'}(t,n) = \delta_{\alpha\beta}\delta_{n,l-l'}\mathrm{e}^{-in\omega t} + \frac{1}{i\hbar}\int_{t_0}^{t} \mathrm{e}^{\frac{i}{\hbar}[\epsilon_\beta-\epsilon+(l-l')\hbar\omega]t_1} \sum_{m=-\infty}^{\infty} \left\langle u_\beta^{m+l'+n}\middle|V(\mathbf{r})\middle|u_\alpha^{m+l}\right\rangle dt_1. \tag{S109}$$

We assume that $t_0 = 0$ and consider different quantum numbers $\alpha \neq \beta$. Then, we can identify that

$$\alpha_{\alpha\beta}^{ll'}(t,n) = -\frac{i}{\hbar}\int_0^t \mathrm{e}^{\frac{i}{\hbar}[\epsilon_\beta-\epsilon+(l-l')\hbar\omega]t_1} \sum_{m=-\infty}^{\infty} \left\langle u_\beta^{m+l'+n}\middle|V(\mathbf{r})\middle|u_\alpha^{m+l}\right\rangle dt_1. \tag{S110}$$

Then, we re-organized the integral by substituting $t_1 = t_1 - t/2$ as follows

$$\alpha_{\alpha\beta}^{ll'}(t,n) = -\frac{i}{\hbar}\mathrm{e}^{-\frac{i}{2\hbar}[\epsilon_\beta-\epsilon+(l-l')\hbar\omega]t}\int_{-t/2}^{t/2} \mathrm{e}^{\frac{i}{\hbar}[\epsilon_\beta-\epsilon+(l-l')\hbar\omega]t_1} \sum_{m=-\infty}^{\infty} \left\langle u_\beta^{m+l'+n}\middle|V(\mathbf{r})\middle|u_\alpha^{m+l}\right\rangle dt_1. \tag{S111}$$

With a considerable long time period, the integral can be described by a delta distribution

$$\lim_{t\to\infty}\int_{-t/2}^{t/2} \mathrm{e}^{\frac{i}{\hbar}\epsilon t_1}\mathrm{d}t_1 = 2\pi\hbar\delta(\epsilon). \tag{S112}$$

Therefore, the scattering amplitude can be expressed as

$$\alpha_{\alpha\beta}^{ll'}(t,n) = -2\pi i\delta(\epsilon_\beta - \epsilon + (l-l')\hbar\omega) \sum_{m=-\infty}^{\infty} \left\langle u_\beta^{m+l'+n}\middle|V(\mathbf{r})\middle|u_\alpha^{m+l}\right\rangle \tag{S113}$$

With the completeness property of free electron eigenstates $|\mathbf{k}\rangle$, we can identify that

$$\alpha_{\alpha\beta}^{ll'}(t,n) = -2\pi i\sum_{\mathbf{k}}\sum_{\mathbf{k}'}\delta(\epsilon_\beta(\mathbf{k}') - \epsilon + (l-l')\hbar\omega) \sum_{m=-\infty}^{\infty} \left\langle u_\beta^{m+l'+n}\middle|\mathbf{k}'\right\rangle \left\langle \mathbf{k}'\middle|V(\mathbf{r})\middle|\mathbf{k}\right\rangle \left\langle\mathbf{k}\middle|u_\alpha^{m+l}\right\rangle. \tag{S114}$$

For a given $\mathbf{k}$ and $\mathbf{k}'$ values we can express the scattering amplitude as follows

$$\alpha_{\alpha\beta}^{ll'}(\mathbf{k},\mathbf{k}',t,n) = -2\pi i V_{\mathbf{k},\mathbf{k}'}\delta(\epsilon_\beta(\mathbf{k}') - \epsilon + (l-l')\hbar\omega) \sum_{m=-\infty}^{\infty} [u_\beta^{m+l'+n}(\mathbf{k}')]^* u_\alpha^{m+l}(\mathbf{k}). \tag{S115}$$

Here, $V_{\mathbf{k},\mathbf{k}'} = \langle\mathbf{k}'|V(\mathbf{r})|\mathbf{k}\rangle$. Then, we introduce a new function

$$c_{\alpha\beta}^{n}(\mathbf{k},\mathbf{k}') = \sum_{m=-\infty}^{\infty} u_\alpha^m(\mathbf{k})[u_\beta^{m+n}(\mathbf{k}')]^*, \tag{S116}$$

and we can present the expression in scattering amplitude in a compact manner

$$\alpha_{\alpha\beta}^{ll'}(\mathbf{k},\mathbf{k}',t,n) = -2\pi i V_{\mathbf{k},\mathbf{k}'}\delta(\epsilon_\beta(\mathbf{k}') - \epsilon + (l-l')\hbar\omega)c_{\alpha\beta}^{l-l'+n}(\mathbf{k},\mathbf{k}'). \tag{S117}$$

Finally, we introduce the transition probability matrix as

$$[A_{\alpha\beta}^{ll'jj'}(\mathbf{k},\mathbf{k}')]_{nn'} = \sum_{\gamma} a_{a\gamma}^{ll'}(\mathbf{k},\mathbf{k}',t,n)[a_{b\gamma}^{jj'}(\mathbf{k},\mathbf{k}',t,n')]^{*}, \tag{S118}$$

and we can obtain

$$[A_{\alpha\beta}^{ll'jj'}(\mathbf{k},\mathbf{k}')]_{nn'} = \\ 4\pi^{2}V_{\mathbf{k},\mathbf{k}'}^{2}\sum_{\gamma} c_{a\gamma}^{l-l'+n}(\mathbf{k},\mathbf{k}')[c_{b\gamma}^{j-j'+n'}(\mathbf{k},\mathbf{k}')]^{*}\delta(\epsilon_{\gamma}(\mathbf{k}')-\epsilon+(l-l')\hbar\omega)\delta(\epsilon_{\gamma}(\mathbf{k}')-\epsilon+(j-j')\hbar\omega) \tag{S119}$$

It important note that we should choose the quasienergies ϵ and any other ϵ_{γ} to be in the central Floquet zone such that

$$|\epsilon-\epsilon_{i}| < \hbar\omega. \tag{S120}$$

This condition alter our derived expression for transition probability matrix into

$$[A_{\alpha\beta}^{ll'jj'}(\mathbf{k},\mathbf{k}')]_{nn'} = 4\pi^{2}V_{\mathbf{k},\mathbf{k}'}^{2}\sum_{\gamma} c_{a\gamma}^{n}(\mathbf{k},\mathbf{k}')[c_{b\gamma}^{n'}(\mathbf{k},\mathbf{k}')]^{*}\delta^{2}(\epsilon-\epsilon_{\gamma}(\mathbf{k}')). \tag{S121}$$

Furthermore, we can re-structure the square of the delta distribution using the following interpretation [11, 12]

$$\delta^{2}(\epsilon) = \delta(\epsilon)\delta(0) = \frac{\delta(\epsilon)}{2\pi\hbar}\lim_{t\to\infty}\int_{-t/2}^{t/2} \mathrm{e}^{\frac{i}{\hbar}0t'}\mathrm{d}t' = \frac{\delta(\epsilon)t}{2\pi\hbar}. \tag{S122}$$

By defining the transition amplitude matrix as

$$\Gamma_{\alpha\beta}^{nn'}(\mathbf{k},\mathbf{k}') = \frac{\mathrm{d}[A_{\alpha\beta}^{ll'jj'}(\mathbf{k},\mathbf{k}')]_{nn'}}{\mathrm{d}t}, \tag{S123}$$

we can evaluate this as follows

$$\Gamma_{\alpha\beta}^{nn'} = \frac{2\pi}{\hbar}V_{\mathbf{k},\mathbf{k}'}^{2}\sum_{\gamma} c_{a\gamma}^{n}(\mathbf{k},\mathbf{k}')[c_{b\gamma}^{n'}(\mathbf{k},\mathbf{k}')]^{*}\delta(\epsilon-\epsilon_{\gamma}(\mathbf{k}')). \tag{S124}$$

To identify the inverse scattering time matrix, first we calculate the impurity average, and then the sum over all momenta over the transition probability matrix

$$\left(\frac{1}{\tau(\epsilon,\mathbf{k})}\right)_{\alpha\beta}^{nn'} = \frac{1}{\mathcal{V}_{\mathbf{k}'}}\sum_{\mathbf{k}'}\left\langle\Gamma_{\alpha\beta}^{nn'}(\mathbf{k},\mathbf{k}')\right\rangle_{\mathrm{imp}}. \tag{S125}$$

Here, $\mathcal{V}_{\mathbf{k}'}$ is the momentum space volume. Since we consider the scattering potential under the Gaussian white noise approximations, we can identify that $\left\langle V_{\mathbf{k},\mathbf{k}'}^{2}\right\rangle_{\mathrm{imp}} = V_{\mathrm{imp}}$ is a constant that only depend on the material [6, 8]. As the final step, we can express the general inverse scattering time matrix as

$$\left(\frac{1}{\tau(\epsilon,\mathbf{k})}\right)_{\alpha\beta}^{nn'} = \frac{2\pi V_{\mathrm{imp}}}{\hbar}\frac{1}{\mathcal{V}_{\mathbf{k}'}}\sum_{\mathbf{k}'}\sum_{\gamma} c_{a\gamma}^{n}(\mathbf{k},\mathbf{k}')[c_{b\gamma}^{n'}(\mathbf{k},\mathbf{k}')]^{*}\delta(\epsilon-\epsilon_{\gamma}(\mathbf{k}')). \tag{S126}$$

This is called as the general Floquet-Fermi golden rule.

2.3 Dressed metal damping factor

In this part of the analysis, we present an expression for the damping factor of conductivity electrons in the dressed metal. Since we are able to explain our natural metallic system using a single energy band, we can neglect the energy band quantum numbers in the general Floquet-Fermi golden rule. Thus, we can represent the inverse scattering time matrix for a given dressed metal as

$$\left(\frac{1}{\tau(\epsilon,\mathbf{k})}\right)^{nn'} = \frac{2\pi V_{\text{imp}}}{\hbar}\frac{1}{\mathcal{V}_{\mathbf{k}'}}\sum_{\mathbf{k}'} c^{n}(\mathbf{k},\mathbf{k}')[c^{n'}(\mathbf{k},\mathbf{k}')]^{*}\delta(\epsilon-\epsilon(\mathbf{k}')), \tag{S127}$$

where

$$c^{n}(\mathbf{k},\mathbf{k}') = \sum_{m=-\infty}^{\infty} u_{m}(\mathbf{k})[u_{m+n}(\mathbf{k}')]^{*}. \tag{S128}$$

Here, $u_n(\mathbf{k})$ is the n-th Fourier component of the Floquet mode. In addition, the disorder is not supposed to change the eigenenergies of the undressed system [6]. Therefore, we can neglect the off-diagonal elements of the inverse scattering time matrix. Finally, we can express the diagonalized inverse scattering time matrix for the dressed metal as follows

$$\left(\frac{1}{\tau(\epsilon,\mathbf{k})}\right)^{nn} = \frac{2\pi V_{\text{imp}}}{\hbar}\frac{1}{\mathcal{V}_{\mathbf{k}'}}\sum_{\mathbf{k}'} |c^{n}(\mathbf{k},\mathbf{k}')|^{2}\delta(\epsilon-\epsilon(\mathbf{k}')). \tag{S129}$$

Applying an upper limit for the dressing field intensity, we identify that non-central element contributions are negligible compared to the central element contribution. Thus, we can approximate the dressed metal inverse scattering time only using the central element ($n = 0$) of the inverse scattering time matrix. In addition, we only focus on the electrons that possess energy near the Fermi energy under the conduction electron movement analysis. Therefore, we can identify the total damping factor for the conducting electrons in the dressed metallic system as

$$\gamma = \frac{1}{\mathcal{V}_{\mathbf{k}}}\sum_{\mathbf{k}}\left(\frac{1}{\tau(\epsilon_F,\mathbf{k})}\right)^{00}\delta\left(\epsilon_F - \frac{\hbar^2 k^2}{2m}\right). \tag{S130}$$

3 Normalized dreesed damping factor for different polarizations

In this section, we try to identify the normalized damping factor of a dressed metal. Here, we consider two different type of polarization type (linear-polarization and circular-polarization) for the dressing field. First, we identify the Floquet state solutions for electrons in dressed metal under these two type of polarization conditions. Then, we derive expression for the normalized damping factor of the dressed metal.

3.1 Wave function solutions for metal under linearly polarized dressing field

We consider a thin metal film placed on the xz-plane of the three-dimensional coordinate space. Furthermore, we assume that the dressing field is a high-intensity linearly polarized electromagnetic field that propagates towards y-direction and perpendicularly to the xz-plane. The dressing field is only applied to the metallic region as given in Fig. S1. Furthermore, we assume that the dressing field consist an x-polarized electric field

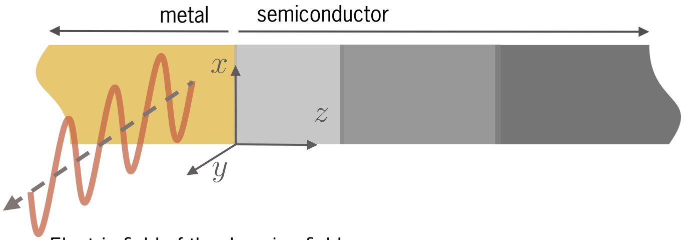

Figure S1: The thin metal film is placed in the xz-plane while the linear-polarized dressing field is applied perpendicular to the xy-plane. Here, the x-polarized electric field component $\mathbf{E}$ *of the dressing field is illustrated in red color.*

$\mathbf{E}(t) = E\sin(\Omega t)\hat{\mathbf{e}}_x$. Here, E is the amplitude of the electric field, Ω is the frequency of the dressing field, and $\hat{\mathbf{e}}_d$ is the unit vector that is pointed to the subscript direction $d = x, y, z$. In addition, we can describe the dressing field using a vector potential with the Coulomb gauge assumption

$$\mathbf{A}(t) = \frac{E}{\Omega}\cos(\Omega t)\hat{\mathbf{e}}_x. \tag{S131}$$

Therefore, the wave function solution of a single electron $\psi(\mathbf{r}, t)$ in the dressed metal should satisfy the time-dependent Schrödinger equation [13]

$$i\hbar\frac{\mathrm{d}\psi(\mathbf{r},t)}{\mathrm{d}t} = \hat{H}_e(t)\psi(\mathbf{r},t), \tag{S132}$$

where

$$\hat{H}_e(t) = \frac{1}{2m}\left[-\hbar^2\nabla^2 + 2ie\hbar\mathbf{A}(t)\cdot\boldsymbol{\nabla} + e^2\mathbf{A}(t)\cdot\mathbf{A}(t)\right]. \tag{S133}$$

Assuming the metal has finite dimensions, we can derive multiple discrete wave function solutions in momentum space for the above time-dependent Schrödinger equation

$$\psi(\mathbf{k},t) = \sqrt{\mathcal{V}}\exp\left[-\frac{i}{\hbar}\left(\tilde{\epsilon}_k t + \frac{e^2E^2}{4m\Omega^2}t - \frac{e\hbar k_x E}{m\Omega^2}\sin(\Omega t) + \frac{e^2E^2}{8m\Omega^3}\sin(2\Omega t)\right)\right]. \tag{S134}$$

Here, $\tilde{\epsilon}_k = \hbar^2k^2/2m$ is the quantized energy levels for a bare electron, $\mathcal{V}$ is the volume of the system, $\mathbf{k} = (k_x, k_y, k_z)^\intercal$ is the wavenumber vector, and $k = |\mathbf{k}|$ with the quantized

values:

$$k = \pi \left[\frac{n_x^2}{L_x^2} + \frac{n_y^2}{L_y^2} + \frac{n_z^2}{L_z^2} \right]^{1/2} \quad \text{with} \quad n_x, n_y, n_z \in \mathbb{Z}^+, \tag{S135}$$

where L_d is the length of the metallic system in the d-direction. Using the Floquet formalism [3, 10], we can express these wave function solutions as Floquet states

$$\psi(\mathbf{k}, t) = \exp\left(-i \frac{\epsilon_k}{\hbar} t \right) u(\mathbf{k}, t). \tag{S136}$$

Here, ϵ_k are the quasienergies, and $u(\mathbf{k}, t)$ are the Floquet modes for each quantized $\mathbf{k}$ values. Thus, we can represent the quasienergies as

$$\epsilon_k = \tilde{\epsilon}_k + \frac{e^2 E^2}{4m\Omega^2}, \tag{S137}$$

and the Floquet modes with

$$u(\mathbf{k}, t) = \sqrt{\mathcal{V}} \exp\left\{ -i \left[\zeta(2\eta k_x) \sin(\Omega t) + \zeta(\eta^2/4) \sin(2\Omega t) \right] \right\}. \tag{S138}$$

Here, $\zeta(\eta) = \hbar\eta/2m\Omega$, and $\eta = -eE/\hbar\Omega$. Using the Jacobi-Anger expansion [14], we can re-formulate the above expression as

$$u(\mathbf{k}, t) = \sum_{n=-\infty}^{\infty} u_n(\mathbf{k}) e^{-in\Omega t}, \tag{S139}$$

where

$$u_n(\mathbf{k}) = \sqrt{\mathcal{V}} J_n \left(\zeta(2\eta k_x), \zeta(\eta^2/4) \right). \tag{S140}$$

Here, $J_l(\cdot)$ are Bessel functions of the first kind with lth integer order. In this simplification, we used the definition of the generalized Bessel function of integer order [14].

3.2 Wave function solutions for metal under circularly polarized dressing field

Now we examine the dressed metal electron behavior under a high-intensity circularly polarized dressing field. We can consider the same system design as the previous subsection, but we replace the linearly polarized dressing field with a circularly polarized dressing field, as given in Fig. S2. Without any loss of generality, we can introduce circu-

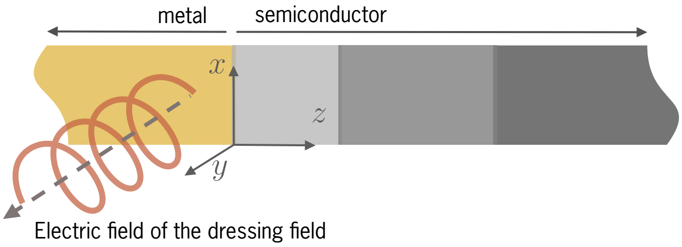

Figure S2: The thin metal film is placed in the xz-plane while the circular-polarized dressing field is applied perpendicular to the xz-plane. Here, the electric field component $\mathbf{E}$ *of the dressing field is illustrated in red color.*

larly polarized dressing field with the following electric field

$$\mathbf{E}(t) = -E\cos(\Omega t)\hat{\mathbf{e}}_x + E\sin(\Omega t)\hat{\mathbf{e}}_z. \tag{S141}$$

Then, we can model the dressing field in the Coulomb gauge as a vector potential

$$\mathbf{A}(t) = \frac{E}{\Omega}\sin(\Omega t)\hat{\mathbf{e}}_x + \frac{E}{\Omega}\cos(\Omega t)\hat{\mathbf{e}}_z. \tag{S142}$$

With the free electron model, the wavefunction of a single electron $\psi(\mathbf{r},t)$ in a metal under a dressing field, should satisfy the time-dependent Schrödinger equation given in equation (S132). Following the same steps as above we can identify the wave function solutions in the momentum space

$$\psi(\mathbf{k},t) = \sqrt{\mathcal{V}}\exp\left[-\frac{i}{\hbar}\left(\tilde{\epsilon}_k t + \frac{e^2E^2}{2m\Omega^2}t - \frac{e\hbar kE\sin(\varphi)}{m\Omega^2}\cos(\Omega t+\vartheta)\right)\right]. \tag{S143}$$

Here, ϑ is the azimuth angle and φ is the polar angle of the given wavenumber $\mathbf{k}$. Then, with the help of the Floquet theory [3, 10], we can identify the quasienergies as

$$\epsilon_k = \tilde{\epsilon}_k + \frac{e^2E^2}{2m\Omega^2}. \tag{S144}$$

In addition, we can represent the Floquet modes by

$$u(\mathbf{k},t) = \sqrt{\mathcal{V}}\exp\left[i\frac{ekE\sin(\varphi)}{m\Omega^2}\cos(\Omega t+\vartheta)\right], \tag{S145}$$

and this can be restructured as

$$\phi(\mathbf{k},t) = \sqrt{\mathcal{V}}\exp[i\Upsilon(k,\varphi)\cos(\Omega t+\vartheta)]. \tag{S146}$$

Here, $\Upsilon(k,\varphi) = \hbar^2\eta k\sin(\varphi)\big/(m\hbar\Omega)$. Finally, we can identify the Floquet states in momentum space as

$$\psi(\mathbf{k},t) = \exp\left(-i\frac{\epsilon_k}{\hbar}t\right)u(\mathbf{k},t), \tag{S147}$$

Calling the Jacobi-Anger expansion [14], we obtain

$$u(\mathbf{k},t) = \sqrt{V}\sum_{n=-\infty}^{\infty} J_n\left(\Upsilon(k,\varphi)\right)e^{in(\vartheta+\pi/2)}e^{in\Omega t}. \tag{S148}$$

Since the Floquet modes are periodic functions in time, we can represent them using the Fourier Series as follows

$$u(\mathbf{k},t) = \sum_{n=-\infty}^{\infty} u_n(\mathbf{k})e^{in\Omega t}, \tag{S149}$$

where

$$u_n(\mathbf{k}) = \sqrt{V}J_n\left(\Upsilon(k,\varphi)\right)e^{in(\vartheta+\pi/2)}. \tag{S150}$$

3.3 Normalized damping factor

Now, we can define the normalized damping factor as

$$\tilde{\gamma} = \frac{\gamma}{\gamma_0}, \tag{S151}$$

where γ_0 is the un-driven ($E = 0$) damping factor of the metal electrons. Then, using the equation (S130), we can present the normalized total damping factor for metal under a linear-polarized dressing field by

$$\tilde{\gamma}_{\text{linear}} = \frac{1}{16\pi^2} \int_0^{\pi} \int_0^{2\pi} \sin\varphi \left[\int_0^{\pi} \int_0^{2\pi} \sin\varphi' J_0^2 \left(\Lambda \left[\sin\vartheta \sin\varphi - \sin\varphi' \sin\vartheta' \right] \right) \mathrm{d}\vartheta' \mathrm{d}\varphi' \right] \mathrm{d}\vartheta \mathrm{d}\varphi, \tag{S152}$$

and the circular-polarized dressing field by

$$\tilde{\gamma}_{\text{circular}} = \frac{1}{4} \int_0^{\pi} \sin\varphi \sum_{m=-\infty}^{\infty} \left[\int_0^{\pi} \sin\varphi' J_m^2 \left(\Lambda \sin(\varphi) \right) J_m^2 \left(\Lambda \sin(\varphi') \right) \mathrm{d}\varphi' \right] \mathrm{d}\varphi. \tag{S1523}$$

Here, $\Lambda = eEk_F \big/ m\Omega^2$, $k_F = \sqrt{2m\epsilon_F}$. For an interesting reader, the detailed simplifications of these expressions can be found in Ref.[13].

References

[1] H. Bruus and K. Flensberg, *Many-body quantum theory in condensed matter physics: an introduction* (OUP Oxford, 2004).

[2] G. D. Mahan, *Many-particle physics* (Springer Science & Business Media, New York, 2000).

[3] M. Holthaus, J. Phys. B: At. Mol. Opt. Phys. **49**, 013001 (2015).

[4] H. Dehghani, T. Oka, and A. Mitra, Phys. Rev. B **91**, 155422 (2015).

[5] V. Lucarini, J. J. Saarinen, K.-E. Peiponen, and E. M. Vartiainen, *Kramers-Kronig relations in optical materials research*, Vol. 110 (Springer Science & Business Media, 2005).

[6] M. Wackerl, P. Wenk, and J. Schliemann, Physical Review B **101**, 184204 (2020).

[7] K. Herath and M. Premaratne, Phys. Rev. B **106**, 235422 (2022).

[8] E. Akkermans and G. Montambaux, *Mesoscopic physics of electrons and photons* (Cambridge University Press, 2007).

[9] J. Rammer, *Quantum transport theory* (CRC Press, 2018).

[10] M. Grifoni and P. Hänggi, Phys. Rep. **304**, 229 (1998).

[11] O. V. Kibis, Europhys. Lett. **107**, 57003 (2014).

[12] K. Dini, O. V. Kibis, and I. A. Shelykh, Phys. Rev. B **93**, 235411 (2016).

[13] K. Herath and M. Premaratne, in *Emerging Imaging and Sensing Technologies for Security and Defence VII*, Vol. 12274 (2022) p. 146.

[14] G. Dattoli, L. Giannessi, L. Mezi, and A. Torre, Il Nuovo Cimento B **105**, 327 (1990).
